# Using biomaterials to improve mesenchymal stem cell therapies for chronic, nonhealing wounds

**DOI:** 10.1002/btm2.10598

**Published:** 2023-09-13

**Authors:** Romina Keshavarz, Sara Olsen, Bethany Almeida

**Affiliations:** ^1^ Department of Chemical and Biomolecular Engineering Clarkson University Potsdam New York USA

**Keywords:** biomaterials, chronic wounds, mesenchymal stem cells, regenerative medicine, tissue engineering, wound healing

## Abstract

Historically, treatment of chronic, nonhealing wounds has focused on managing symptoms using biomaterial‐based wound dressings, which do not adequately address the underlying clinical issue. Mesenchymal stem cells (MSCs) are a promising cell‐based therapy for the treatment of chronic, nonhealing wounds, yet inherent cellular heterogeneity and susceptibility to death during injection limit their clinical use. Recently, researchers have begun to explore the synergistic effects of combined MSC‐biomaterial therapies, where the biomaterial serves as a scaffold to protect the MSCs and provides physiologically relevant physicochemical cues that can direct MSC immunomodulatory behavior. In this review, we highlight recent progress in this field with a focus on the most commonly used biomaterials, classified based on their source, including natural biomaterials, synthetic biomaterials, and the combination of natural and synthetic biomaterials. We also discuss current challenges regarding the clinical translation of these therapies, as well as a perspective on the future outlook of the field.

**Abbreviations**
ADH: adipic acid dihydrazide
ADM: acellular dermal matrix
ADSCs: adipose‐derived stem cells
AgNPs: silver nanoparticles
AM: amniotic membrane
AuNPs: gold nanoparticles
bFGF: basic fibroblast growth factor
BMSCs: bone marrow‐derived stem cells
Col: collagen
CR: contraction rate
DAB: 3,3′‐diaminobenzidine
DAM: decellularized adipose matrix
DDM: decellularized dermal matrix
DF‐PU: difunctional polyurethane
DFU: diabetic foot ulcer
DM: diabetes mellitus
ECM: extracellular matrix
EDC: 1‐ethyl‐3‐(3‐(dimethylamino)propyl)carbodiimide hydrochloride
EGF: epidermal growth factor
ER: epithelialization rate
FDA: food and drug administration
GA: glutaraldehyde
GAG: glycosaminoglycan
GFP: green fluorescent protein
GO: graphene oxide
HA: hyaluronic acid
HA‐ALD: hyaluronic acid‐aldehyde
H&E: hematoxylin and eosin
IFN‐γ: interferon‐gamma
IL: interleukin
MPs: microparticles
MSC: mesenchymal stem cell
*N*‐chitosan: *N*‐carboxyethyl chitosan
PBS: phosphate‐buffered saline
PEFs: polyethyleneimine‐modified polycaprolactone fibers
PEG: poly(ethylene glycol)
PF‐127: pluronic F‐127
PGE_2_
: prostaglandin E_2_
Pul: pullulan
PVA: polyvinyl alcohol
RAS: radially aligned scaffolds
RGD: arginyl‐glycyl‐aspartic acid
RGONPs: reduced graphene oxide nanoparticles
SAP: sodium ascorbyl phosphate
SIS: small intestinal submucosa
STZ: streptozotocin
TGF: transforming growth factor
TNF‐α: tumor necrosis factor‐α
UCMSCs: umbilical cord‐derived mesenchymal stem cells
UV: ultraviolet
VAS: vertically aligned scaffolds
VEGF: vascular endothelial growth factor
WJMSC: Wharton's jelly mesenchymal stem cell
β‐GP: β‐glycerophosphate
2D: two‐dimensional
3D: three‐dimensional

**Translational Impact Statement**

Chronic, nonhealing wounds affect millions of people worldwide wherein the body is incapable of self‐healing. Currently, doctors are focused primarily on mitigating and managing symptoms rather than treating the underlying disease. In this review, we explore recent advances in the literature on the use of mesenchymal stem cells loaded in various types of biomaterials as a potential therapy for chronic, nonhealing wound treatment. We also provide a forward perspective of the field, exploring how researchers might address these challenges and achieve clinical translation.

## 1 INTRODUCTION

Chronic, nonhealing wounds are full‐thickness lesions stemming from a range of diseases, such as diabetes and obesity, that persist for many months or years.^1^, ^2^ There are many different types of chronic wounds, including ulcers (e.g., diabetic, venous, arterial, pressure),^3^ pressure sores,^4^ burns (e.g., radiation, surgical),^3^ keloids,^4^ hypertrophic scars,^4^ and fibrosis.^5^ An estimated 1%–2% of the world's population will suffer from a disease‐related chronic, nonhealing wound in their lifetime.^1^ Due to their increasing prevalence and severity, chronic wounds are becoming a major public health burden. Recent Medicare cost projections estimate expenditures of $28 to $97 billion on the management and treatment of chronic wounds in the US alone.^2^, ^6^

**FIGURE 1:**
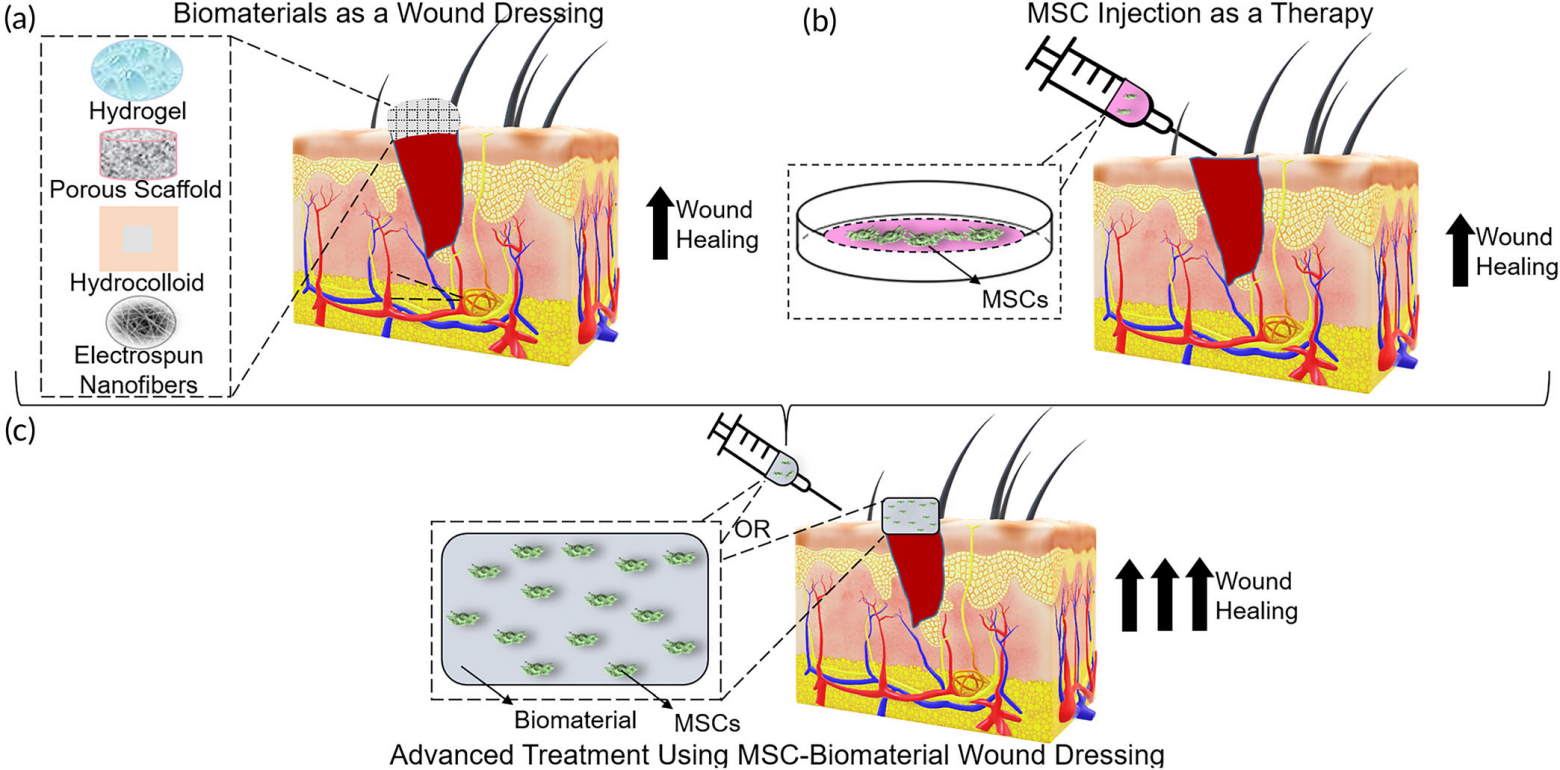
Schematic of the different treatment methods of chronic wounds, including (a) biomaterial wound dressings, such as hydrogels, porous scaffolds, hydrocolloids, and electrospun nanofibers, to name a few, with biocompatible and tunable properties which can be combined with bioactive factors, (b) mesenchymal stem cells (MSCs) that are capable of secreting paracrine factors and modulating immune cells, and (c) a combinatorial approach using biomaterials in conjunction with MSCs, whereby the biomaterial acts as a carrier for MSCs delivery or serves to improve their therapeutic efficacy.

The most common approach for treating chronic wounds is the application of biomaterials, typically as wound dressings, which serve to hydrate and protect the wound and may be designed to release anti‐inflammatory or other wound healing factors (Figure 1a).^7^, ^8^, ^9^ However, treatment using wound dressings has focused on managing symptoms rather than addressing the underlying problem, resulting in more than 50% of treatments failing.^10^ As a result, persisting chronic wounds often end up in surgical intervention (e.g., graft implantation) or even amputation.^4^, ^11^ Thus, there is an urgent need to develop therapies for the treatment of chronic wounds.

Stem cells have recently garnered interest as a potential therapy for chronic wounds; mesenchymal stem cell (MSC) injection, in particular, is extensively explored due to the significant role of MSCs in wound healing (Figure 1b).^12^, ^13^, ^14^ It should be noted that MSCs can also be referred to as mesenchymal stromal cells, the latter usually referring to the immunomodulatory properties and cytokine excretion of stromal cells. These definitions are used interchangeably throughout this review depending on the use of the original paper being discussed. MSCs have been shown to boost angiogenesis, decrease inflammation, increase re‐epithelialization and granulation tissue formation, influence extracellular matrix (ECM) remodeling, and speed up wound closure through the secretion of paracrine factors.^15^, ^16^, ^17^, ^18^ MSCs can also modulate immune cell function. For example, MSCs are capable of inhibiting activation, differentiation, and maturation of natural killer cells, dendritic cells, T‐cells, and B‐cells, as well as inducing macrophage polarization from the pro‐inflammatory (M1) to anti‐inflammatory (M2) phenotype.^13^, ^19^, ^20^

Despite their potential, there remains many challenges that have affected the clinical use of MSC‐based therapies. The first is MSC heterogeneity, where MSCs from different tissue sources and donors may have different immunomodulatory potentials.^15^ In addition, the immunomodulation efficacy of MSCs is known to decrease in chronic wounds because of persistent inflammation, which affects cytokine expression levels and increases the presence of inflammatory cells (e.g., M1 macrophages).^19^, ^21^ Furthermore, MSCs are typically expanded in vitro prior to transplantation, yet there are significant differences between the in vitro and in vivo microenvironment that affect MSC differentiation, proliferation, and immunomodulatory potential.^22^ Transplanted MSCs also face immune rejection,^21^ poor engraftment,^16^ and low cell survival, often due to oxidative stress from hypoxia^23^ and shear forces from the injection.^21^

To mitigate the challenges of using biomaterials or MSCs individually as therapies for chronic wounds, recent literature has focused on combining MSCs with a range of biomaterials to improve chronic wound healing outcomes (Figure 1c). Biomaterials show immunoprotective function by acting as barriers to prevent immune cells from attacking and protect the cells from host inflammation. Therefore, immunomodulatory biomaterials could facilitate the regeneration process.^24^ Although biochemical signals, such as synthetic drugs or small molecules, may be added to the MSCs during in vitro expansion and transplantation to improve their immunomodulatory potential,^25^, ^26^ chronic wounds contain proteases that degrade these biochemical molecules and reduce their efficacy.^27^ Furthermore, biochemical signals do not protect the MSCs from damage due to transplantation. In turn, biomaterial scaffolds can provide a three‐dimensional (3D) microenvironment that supports MSC adhesion, growth, and retention.^28^ For example, through forming a lubricating layer at the edges of the syringe, injectable shear‐thinning biomaterials trigger almost equal velocities at the edges and the center of the syringe, diminishing the shear forces applied to cells during the injection.^29^ Moreover, to hinder cell damage from extreme oxidative stress in chronic wounds, biomaterials with antioxidant activity, such as cysteine and ulvan, can be used to downregulate the excessive production of reactive oxygen species.^30^, ^31^

In addition, the physicochemical properties of the biomaterial itself may regulate MSC behavior to improve immunomodulatory paracrine factor secretion and accelerate healing.^32^, ^33^, ^34^, ^35^ For example, physicochemical properties such as matrix porosity,^34^ topography (i.e., fiber alignment, surface roughness, and surface structure),^34^, ^36^, ^37^ matrix stiffness,^34^, ^38^ matrix viscoelasticity,^33^, ^38^ hydrophobicity,^39^ and surface charge^39^ have all been shown to direct MSC differentiation and immunomodulation. Furthermore, biomaterials may serve as delivery vehicles for biochemical drugs and small molecules capable of modulating MSC behavior,^21^, ^36^ and encapsulation of biochemical drugs and small molecules in biomaterials may protect these biochemical signals from degradation. For example, using oxygen‐releasing biomaterials, made by incorporating calcium peroxide into biomaterials, can increase cell survival under hypoxia (reduced oxygen concentration) conditions in chronic wounds, leading to expedited wound healing.^40^

In this review, we highlight the recent progress from roughly the past 5 years made in combined MSC‐biomaterial therapies for treating chronic, nonhealing wounds. In the first part of the review, we give a detailed overview of the current preclinical literature, which is subdivided by source of biomaterial. The second part of the review takes a forward look at the field, starting with detailing the current clinical advances of MSC‐biomaterial therapies, as well as discussing the remaining challenges in the literature and perspectives on future directions for the field.

## 2 MSC‐BIOMATERIAL THERAPIES FOR TREATING CHRONIC, NONHEALING WOUNDS

The synergistic effects of biomaterials and MSCs on treating chronic, nonhealing wounds have the potential to lead to a breakthrough in wound healing. Thus, there has been considerable interest in developing MSC‐biomaterial therapies capable of overcoming the limitations of biomaterials or MSCs alone. Perhaps the most commonly employed biomaterials for MSC‐biomaterial therapies to treat chronic, nonhealing wounds are polymeric biomaterials. Polymeric wound dressings are widely used for their ability to provide a moist environment,^41^ cover a great part of the wound surface,^42^ stimulate growth factors,^43^ boost vascularization,^44^ and preserve the wound site from infection.^45^ In addition, polymers are extensively used with MSCs due to their innate biocompatibility^46^ and highly tunable physicochemical properties,^47^ promoting the design of highly functional MSC‐biomaterial systems. Polymeric biomaterials can take the form of scaffolds,^48^, ^49^ films,^50^ hydrocolloids,^51^ hydrogels,^52^, ^53^, ^54^, ^55^, ^56^, ^57^ hydrofibers,^58^ and electrospun nanofibers.^59^ Among these, hydrogels are widely preferred due to their superior properties, such as biodegradability,^60^ high water content retention,^60^, ^61^ easy fabrication,^62^ high capacity for drug loading,^63^ immunomodulation,^64^ and their ability to boost MSC immunomodulatory properties.^65^ In this section, we have summarized the most common polymeric biomaterials based on their source which can be natural, synthetic, or the combination of natural and synthetic polymers.

### 2.1 Natural biomaterials

Among the different naturally occurring polymers, collagen (Col),^66^ fibrin,^23^ hyaluronic acid (HA),^67^ gelatin,^68^ chitin^69^ and chitosan,^70^, ^71^ and decellularized ECM‐derived scaffolds^72^, ^73^ are commonly used in combination with MSCs for wound healing applications. Col, one of the main structural proteins in ECM, enhances MSC adhesion, survival, and proliferation.^74^ A Col scaffold, fabricated using type 1 rat tail Col added to phosphate‐buffered saline (PBS) and 10× Dulbecco's modified eagle medium, was seeded with either murine adipose‐derived stem cells (ADSCs) or bone marrow‐derived stem cells (BMSCs).^66^ This cell‐laden scaffold was used to treat a splinted‐full thickness excisional wound in diabetic C57BL/6 mice. Wounds with the ADSC‐seeded Col scaffold and BMSC‐seeded Col scaffold were shown to have similar accelerated rates of wound closure, approximately 1.4‐fold, compared to the acellular control after 5 days.

Gelatin is a protein derived from Col containing arginyl‐glycyl‐aspartic acid (RGD) peptides that is easier to handle compared to Col.^75^ Lu et al. investigated the effectiveness of human ADSCs (as spheroids or suspensions) encapsulated in a gelatin hydrogel on wound healing in a Wistar rat burn wound model.^68^ The combination of ADSCs and gelatin led to expedited wound healing. The ADSC spheroids + gelatin showed a 1.2‐, 1.5‐, 1.7‐, and 1.8‐fold higher wound healing rate after 14 days in comparison with an ADSC suspension + gelatin, gelatin, ADSC suspension, and the nontreated group, respectively. CD31 staining revealed that culture in spheroids increased secretion of growth factors, namely platelet‐derived growth factor, vascular endothelial growth factor (VEGF), and basic fibroblast growth factor (bFGF), as well as enhanced angiogenesis through cell–cell and cell‐ECM contacts. In addition, hematoxylin and eosin (H&E) staining showed increased tissue regeneration; the ADSC spheroids + gelatin group had a significantly thicker epidermal layer (~1.6‐fold) than the ADSC suspension + gelatin group after 14 days, whereas the thickness was the same for the other groups.

**FIGURE 2:**
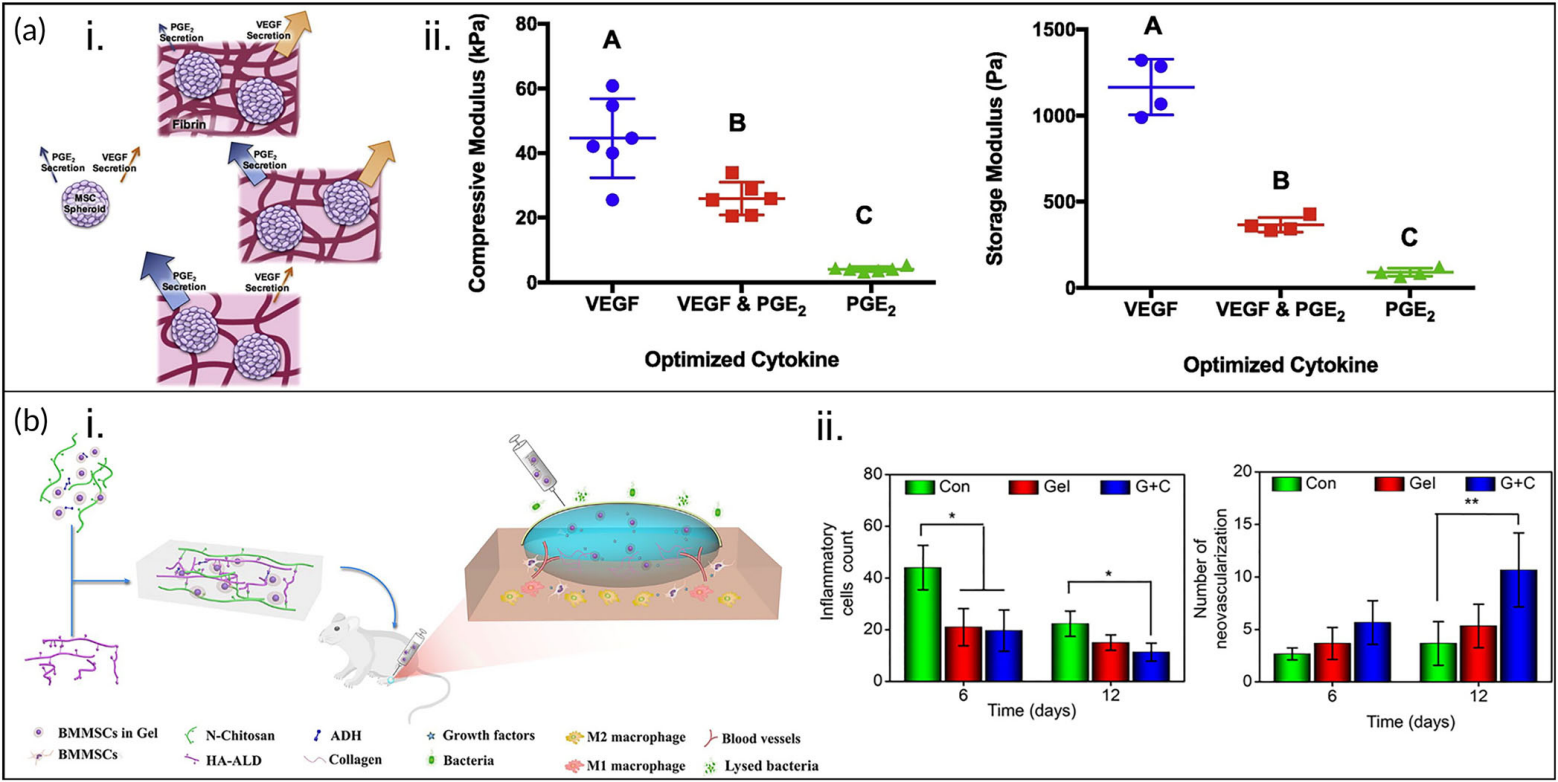
Natural polymer‐mesenchymal stem cell (MSC) therapies to enhance wound healing. (a) (i) Schematic representation of MSC spheroids alone or encapsulated in fibrin hydrogels with varying fibrin densities, demonstrating the effects of encapsulation within the hydrogel and hydrogel properties on vascular endothelial growth factor (VEGF) and prostaglandin E_2_ (PGE_2_) secretion. (ii) Compressive moduli (left) and shear storage moduli (right) of fibrin hydrogels, demonstrating that intermediate hydrogel properties resulted in optimized cytokine secretion (the highest simultaneous secretion of VEGF and PGE_2_). An increase in the compressive and storage moduli resulted in increasing VEGF secretion and maintaining PGE_2_ secretion, thereby enhancing angiogenesis and reducing inflammation. All groups were statistically significant (*p* < 0.05). Reprinted from Reference 23, Copyright (2017), with permission from Elsevier. (b) (i) Schematic of *N*‐carboxyethyl chitosan (*N*‐chitosan)/hyaluronic acid‐aldehyde (HA‐ALD) hydrogel + BMSCs. ADH, adipic acid dihydrazide. (ii) The hydrogel + BMSCs group (G + C) significantly improved wound healing by reducing chronic inflammation and increasing angiogenesis (*p < 0.05, **p < 0.01). Con, control group; Gel, hydrogel group. Reprinted from Reference 70, Copyright (2020), with permission from the authors.

Another commonly used natural polymer is fibrin, a fibrous protein often used in blood clotting, known to form gels with tunable physical properties.^76^ Murphy et al. designed a fibrin hydrogel encapsulating MSC spheroids that was capable of upregulating the secretion of trophic factors, thereby boosting angiogenesis, modulating inflammation, and consequently enhancing wound healing (Figure 2a).^23^ Briefly, this hydrogel was fabricated by mixing fibrinogen, thrombin, NaCl, CaCl_2_, and aprotinin in PBS. Using a multifactorial Box–Behnken statistical method, the researchers developed hydrogels with tunable stiffness and degradation rate by changing hydrogel composition. This allowed the researchers to control the simultaneous secretion of VEGF and prostaglandin E_2_ (PGE_2_) from the MSC spheroids. They found that hydrogels with high fibrinogen (20 mg/mL), low CaCl_2_, and high NaCl concentrations demonstrated high stiffness (~40 kPa) and the highest VEGF secretion. However, hydrogels with low fibrinogen (5 mg/mL) but still low CaCl_2_ and high NaCl concentrations resulted in softer hydrogels and the highest PGE_2_ secretion. Therefore, intermediate mechanical properties were capable of high secretion of both VEGF and PGE_2_ simultaneously.

HA, a nonsulfated glycosaminoglycan (GAG) abundant in the ECM, is commonly investigated due to its ability to readily form hydrogels and control cell signaling.^77^ Gómez‐Aristizábal et al. investigated the immunomodulatory effects of MSCs as a function of the molecular weight of the HA used (1.6 MDa, 150 kDa, or 7.5 kDa).^67^ Human BMSCs were incubated with the different HA molecular weights for 1, 4, and 24 h. The researchers found no significant change in gene expression of the MSCs with respect to HA molecular weight. Furthermore, the addition of 1.6 MDa HA with MSCs resulted in a decrease in MSC‐mediated inhibition of activated T‐helper cell proliferation, which was not altered by the addition of interferon‐gamma (IFN‐γ), a pro‐inflammatory molecule known to influence MSC immunomodulation, as well as an increase in the frequency of M2 monocyte‐derived macrophages. Similar results were seen with respect to interleukin (IL)‐10 levels and IL‐2 secretion, thereby demonstrating that high molecular weight HA improves the anti‐inflammatory effects of MSCs.

Chitin, the second most abundant natural polysaccharide, is another potential biomaterial for chronic wound healing because they are suitable for immobilization of MSCs.^18^ In a study by Liu et al., squid‐extracted β‐chitin nanofibers ranging from 5 to 10 nm in diameter were fabricated into a hydrogel by adding cell culture medium to the β‐chitin nanofibers dispersion at a ratio of 1:3, encapsulated with C57BL/6 mouse ADSCs, and investigated in Sprague–Dawley rats.^69^ The researchers found that the ADSC‐loaded, β‐chitin nanofiber hydrogel increased the wound healing rate 3.5‐fold compared with the control, ADSCs alone, and hydrogel alone groups after just 2 days of treatment. Furthermore, they elucidated that this accelerated wound‐healing process was obtained by controlling the transforming growth factor (TGF)‐β/smad3 signaling pathway.

By combining different natural polymers, researchers are able to fabricate advanced, bioinstructive biomaterials. Some researchers currently employ a dual crosslinking approach to decrease gelation time and enhance mechanical properties.^78^ For example, Vining et al. investigated a hydrogel comprised of fibrillar type I Col networks and functionalized alginate crosslinked using both ionic and covalent crosslinking (norbornene‐tetrazine click chemistry) methods.^38^ The use of a covalent, secondary alginate crosslinking allowed the researchers to tune the hydrogel viscoelasticity and stiffness to control the MSCs' immunomodulatory behavior. Furthermore, the sequential combination of ionic and covalent crosslinking methods between alginate and type I Col promoted the self‐assembly of a fibrillar Col structure that mimics the native ECM while also decreasing gelation time and increasing the mechanical properties of the gel. They found that human BMSC gene expression of cyclo‐oxygenase‐2 and tumor necrosis factor‐α (TNF‐α)‐stimulated gene‐6 were upregulated in this hydrogel compared to BMSCs on tissue culture plastic. Furthermore, increasing hydrogel stiffness (0.25, 0.5, and 2.5 kPa) resulted in increased upregulation of these genes. For example, a 10‐fold higher expression of TNF‐α‐stimulated gene‐6 in a hydrogel with 0.25 kPa stiffness increased to more than a 40‐fold higher expression of TNF‐α‐stimulated gene‐6 in a hydrogel with 2.5 kPa stiffness.

In another study, Bai et al. investigated the efficacy of BMSC‐loaded HA‐chitosan hydrogels on modulating chronic inflammation caused by a diabetic foot ulcer (DFU) in a diabetic Sprague–Dawley rat model (Figure 2b).^70^ Briefly, aldehyde‐functionalized HA was crosslinked with *N*‐carboxyethyl chitosan via an adipic acid dihydrazide crosslinker using bioorthogonal click chemistry to form an injectable, self‐healing hydrogel loaded with BMSCs. After 9 days, TGF‐β1 and VEGF secretions for the BMSCs‐loaded hydrogel were both enhanced by 24% compared to the hydrogel‐only group and increased by 36% and 31% compared to the untreated group, respectively. Similarly, bFGF secretion of the BMSCs‐loaded hydrogel was ~1.5‐fold higher than the control group 9 days post‐treatment. Furthermore, the BMSCs‐loaded hydrogel reduced macrophage inflammation, confirmed by a 31% decrease in CD86 expression and a 1.5‐fold increase in CD163 expression, compared to the control group at Day 6. Granulation tissue formation, Col deposition, and angiogenesis were all increased, as well, indicating DFU healing. In a different study by Hsu et al., adult ADSC spheroids were injected onto wounds and covered by HA hydrogel embedded in a chitosan‐grafted scaffold.^71^ The composite hydrogel with ADSC spheroids had increased angiogenesis and wound closure percentage compared with the hydrogel without ADSCs or with single ADSCs. The researchers determined that this was due to the ability of the composite hydrogel to preserve the ADSCs' spheroid morphology; spheroid morphology and high cell densities are known to increase paracrine secretion.

Similarly, Shukla et al. developed an MSC‐loaded hydrogel comprised of chitosan and gelatin to treat surgical wounds studied on goat models, which they call Velgraft®.^79^ They found that the combined chitosan‐gelatin hydrogel encapsulating MSCs resulted in complete re‐epithelialization, increased deposition of Col, enriched blood vessels, and renewal of both sebaceous glands and hair follicles 28 days post‐treatment compared with Soframycin‐treated and sham‐operated groups. The researchers discuss that these results were due to higher expressions of VEGF, CD31, and TGF‐β1 in the Velgraft®‐treated group. Also, the wound contraction after 28 days was 1.3‐fold higher in the Velgraft®‐treated group compared to the Soframycin‐treated and control groups. In another study, Yang et al. fabricated an injectable, thermosensitive, chitosan/Col/β‐glycerophosphate (β‐GP) hydrogel loaded with MSC spheroids by incubation of the mixed solution at 37 °C.^57^ They demonstrated that the MSC spheroid‐loaded hydrogel had faster wound closure in a DB/DB diabetic mouse model due to a 2.6‐ and 1.8‐fold increase in angiogenesis compared with nonspheroidal MSCs injected alone or a chitosan/Col/β‐GP hydrogel loaded with nonspheroidal MSCs, respectively.

Other types of excellent candidates as natural biomaterials for the MSC‐mediated treatment of chronic, nonhealing wounds are decellularized ECM‐derived scaffolds. Depending on the tissue source, the decellularized ECM may contain growth factors and molecules beneficial for chronic wound healing.^80^ Furthermore, their innate biocompatibility and mimicry of native tissue allow decellularized ECM scaffolds to serve both as cell delivery vehicles and bioactive biomaterials to direct cell behavior.^80^ Other advantages include that these scaffolds can maintain their native physicochemical properties and complex 3D structure after decellularization, degradation does not release toxic by‐products, and, depending on the tissue source, they can mitigate an immunogenic response and work synergistically with MSCs to modulate macrophage polarization.^80^, ^81^

**FIGURE 3:**
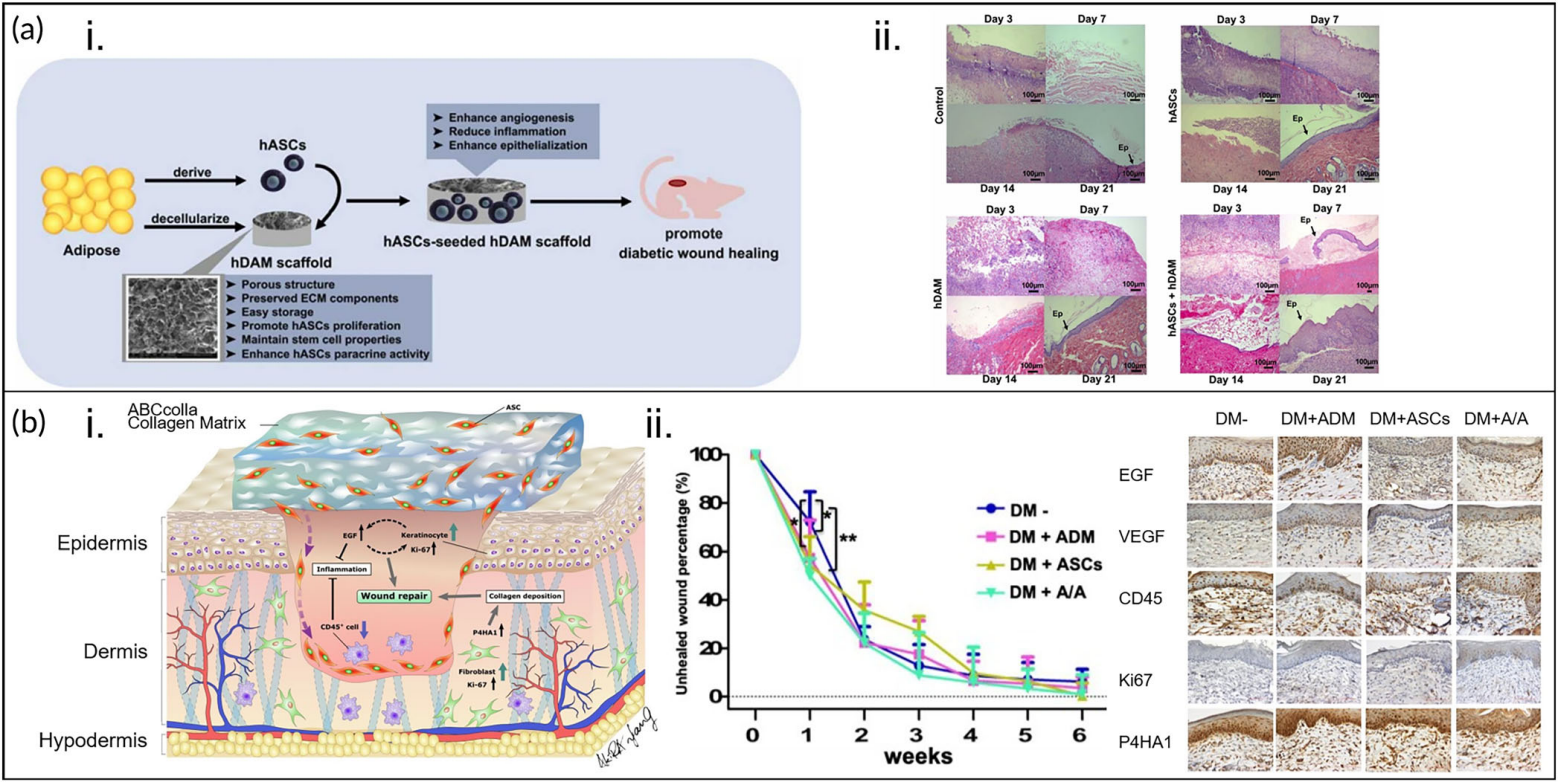
Decellularized tissue‐derived biomaterial‐MSC therapies to improve chronic wound healing. (a) (i) Schematic of the adipose‐derived stem cells (ADSC) + decellularized adipose matrix (DAM) wound dressing. (ii) Hematoxylin and eosin (H&E) histological staining of the control, ADSCs, DAM, and ADSCs + DAM groups, showing better epidermis formation in the ADSCs + DAM group. Scale bars = 100 μm. Reprinted from Reference 72, Copyright (2022), with permission from Elsevier. (b) (i) Illustration of the ADSC‐loaded ABCcolla® collagen (Col) matrix from a decellularized dermal matrix (DDM), improving fibroblasts and keratinocyte proliferation, Col deposition, and modulating inflammation. (ii) Accelerated wound healing in the diabetes mellitus (DM) + A/A group (DM with ADSC‐DDM) (*p < 0.05, **p < 0.01). (iii) Immunohistochemistry images of epidermal growth factor (EGF), vascular endothelial growth factor (VEGF), CD45, Ki67, and prolyl 4‐hydroxylase (P4HA1) for DM—(DM without treatment), DM + DDM, DM + ADSCs, and DM + A/A samples. Scale bars = 50 μm. Reprinted from Reference 73, Copyright (2020), with permission from the authors.

The most commonly used source of decellularized ECM‐derived scaffolds is human tissue. Although more cost‐effective and readily available, the microenvironment of animal‐derived tissue is often significantly different from that of a human, particularly in immunological, wound healing applications.^82^ Adipose matrix is a particularly common tissue source as it is easily obtained as a waste product from liposuction.^83^ In one study by Chen et al., a human decellularized adipose matrix (DAM)‐derived hydrogel was produced through five steps: decellularization, lyophilization, grinding, pepsin digestion, and pH neutralization.^84^ Human ADSCs were added to the DAM pre‐gel liquid solution before gelation. Using a diabetic, full‐thickness wound on a KK/Upj‐Ay/J mouse model, the researchers demonstrated enhanced neovascularization and wound closure 7 days post‐treatment, as well as enhanced quality of regeneration after 14 days compared with the hydrogel‐ and ADSC‐only controls. The authors elucidated that this enhanced wound healing was achieved through increased secretion of hepatocyte growth factors. In another study by the same research group, the researchers tested an alternative preparation method.^72^ Human adipose tissue was decellularized, lyophilized, and sterilized using 70% ethanol. Human ADSCs were seeded in the DAM scaffold (Figure 3a). Diabetic Wistar rats with full‐thickness wounds were treated with this ADSC‐seeded DAM scaffold and demonstrated enhanced angiogenesis through increased VEGF secretion. H&E staining showed that there was increased epithelization for the ADSC‐seeded DAM scaffold compared with the untreated group and reduced inflammatory cytokine expression after 7 days. Furthermore, regeneration of skin appendages occurred after 21 days for the ADSC‐seeded DAM scaffold but not the untreated group.

Another commonly used human tissue source is the amniotic membrane (AM), which secretes pro‐angiogenic, anti‐inflammatory, anti‐fibrotic, and anti‐bacterial growth factors, and cytokines.^85^ They are readily available in various forms, such as AM powder and decellularized AM, which make them cost‐effective scaffolds for stem cell culture.^85^ Hashemi et al. developed human AM scaffolds onto which dermal fibroblasts and Wharton's jelly mesenchymal stem cells (WJMSCs) were co‐cultured.^86^ This scaffold was prepared by isolating the tissue from the chorionic layers and decellularizing by using 0.25% trypsin–EDTA. The efficacy of the cell‐laden AM scaffold was tested against a human type II diabetic ulcer model, and the researchers found 81% and 94% wound healing after 6 and 9 days, respectively, resulting in complete regeneration after 9 days of treatment.

Despite the inherent benefits of using human tissue, animal tissue remains a popular source for decellularized matrices. Porcine tissue is the most prevalent, as porcine models are considered the most similar to human.^87^ Jiang et al. investigated a human ADSC‐seeded porcine small intestinal submucosa (SIS) scaffold on a streptozotocin (STZ)‐induced type 2 diabetic Sprague–Dawley rat model.^88^ This cell‐laden biomaterial, prepared by decellularization, lyophilization, and sterilization using ethylene oxide, was found to synergistically improve wound healing. The SIS scaffold triggered an increase in the secretion of growth factors necessary for wound healing, including epidermal growth factor (EGF), TGF‐β, bFGF, and VEGF from the ADSC‐seeded scaffold compared with ADSCs alone. In vivo, this synergy was further demonstrated by faster and better‐quality healing post‐treatment with the SIS scaffold + ADSCs compared to the controls, blank (PBS), ADSCs‐only, and SIS‐only. For the SIS scaffold + ADSCs group, there was a 1.9‐ and 1.8‐fold increase in angiogenesis over 21 days compared to the PBS and the ADSC‐only groups, respectively, with a subsequent increase of 1.5‐fold in blood vessel maturation after 28 days compared to both PBS and the SIS‐only groups. In addition, compared with the PBS‐only group, the ADSC‐laden SIS scaffold demonstrated a 44% increase in macrophage infiltration up to Day 14 followed by a 140% and 300% decrease at Days 21 and 28, which follows a typical pattern for wound healing in nonchronic wounds, demonstrating healing of the diabetic ulcer.

In another study by Chou et al., a porcine skin‐derived dermal matrix (DDM) decellularized by supercritical carbon dioxide was loaded with rat ADSCs, and its efficacy was examined in STZ‐induced Wistar rats (Figure 3b).^73^ The researchers showed that the ADSC‐laden DDM expedited diabetic wound healing by reducing inflammation (71% decrease in CD45 expression compared with the untreated diabetic wound), promoting re‐epithelialization (32% increase in EGF expression compared with the untreated diabetic wound), increasing angiogenesis (20% increase in VEGF expression compared with the diabetic wound treated with DDM‐only), increasing Col synthesis and deposition (50% increase in prolyl 4‐hydroxylase expression compared with the untreated diabetic wound), and increased cell proliferation (85% increase in Ki67 expression compared with the untreated diabetic wound). They determined that the presence of the Col‐rich ECM in the DDM contributed to this enhanced healing by influencing ADSC proliferation, regeneration, and paracrine signal expression.

Murine and ovine decellularized tissues have also been explored in the literature. Chu et al. fabricated a green fluorescent protein (GFP)‐tagged mouse skin DDM, prepared by decellularization with Triton X‐100 and trypsin followed by sterilization using chloroform and methanol.^89^ The DDM was loaded with C57BL/6 mouse MSCs and tested against the STZ‐induced diabetic ICR mouse model. The researchers observed that DDM‐MSC scaffolds increased angiogenesis, re‐epithelialization, and wound closure compared with untreated control and DDM alone groups. Furthermore, GFP tagging allowed the researchers to use multiphoton microscopy to simultaneously track the synthesis of Col type I fibers and MSC activity during wound healing with second harmonic generation imaging and 2‐photon excitation fluorescence imaging, respectively. In a different study by Rashtbar et al., a decellularized ovine SIS scaffold seeded with rat BMSCs completely healed a critical‐sized, full‐thickness wound in Wistar and Lewis rats within 21 days compared with the untreated and SIS scaffold groups alone.^90^ Furthermore, H&E staining of the BMSC‐seeded SIS scaffold showed an increase in re‐epithelialization compared to the untreated wound, as well as the most regeneration of skin appendages. The researchers suggest that this may be due to the immunomodulatory potential of BMSCs and their ability to differentiate into fibroblasts and keratinocytes.

Yet other studies have sought to combine naturally occurring polymers with decellularized tissue scaffolds in order to harness the properties of both. Bo et al. investigated a platform combining a polymer and a decellularized tissue to develop a porcine skin DDM/nitrobenzene‐modified HA hydrogel loaded with ADSCs.^91^ This hydrogel was fabricated by exposing the HA and DDM in situ to 365 nm ultraviolet (UV) irradiation for 90 s. Nude mice with full‐thickness regularly and irregularly (triangular, L‐shaped, and trapezoidal) shaped wounds were treated with the hydrogel + ADSC, hydrogel‐only, ADSC‐only, and PBS‐treated (control) group. The hydrogel + ADSC group showed expedited healing of irregularly shaped wounds; the ADSC‐seeded DDM/HA hydrogel demonstrated a ~1.9‐fold increased neovascularization and ~2.7‐fold increased Col deposition when compared to the control after 21 days.

However, the most common approach is the combination of nanoparticles with macroscale, 3D scaffolds made from naturally sourced polymeric biomaterials, resulting in the generation of multi‐functional biomaterials. Nanobiomaterials have a wide range of physicochemical properties based on their materials and structures that can significantly vary from bulk macroscale properties. For example, colloidal solutions of gold nanoparticles (AuNPs) are known to have varying optical properties (i.e., change colors) depending on the nanoparticle diameter.^92^ Moreover, the intrinsic properties of nanoparticles can act on MSCs to improve cellular function, such as iron oxide nanoparticles, which have been shown to improve adipogenesis and osteogenesis of MSCs, just by their presence,^93^ or they may encapsulate drugs or other small molecules that can work synergistically with the MSCs and improve immunomodulatory outcomes.^94^ This combination allows encapsulated cells to receive signals from the macroscale scaffold, the nanoparticle, and any encapsulated biochemical signals.

**FIGURE 4:**
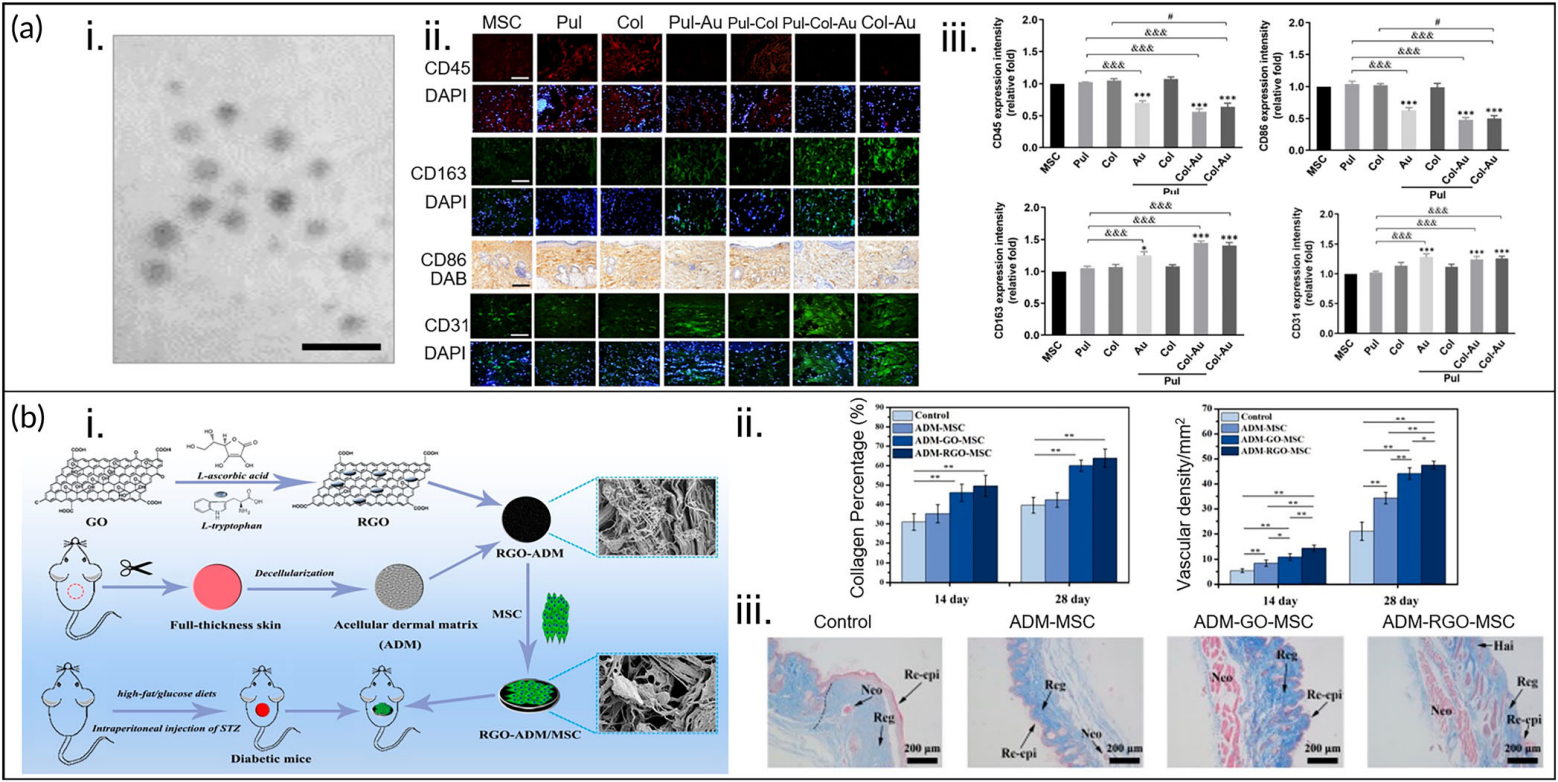
Nanoparticle‐loaded, naturally dervied biomaterials promote the chronic wound‐healing potential of MSCs. (a) (i) Scanning electron microscopy of the pullulan‐collagen‐gold nanoparticles (Pul‐Col‐AuNPs) nanocomposite. Scale bar = 20 nm. (ii) Immunohistochemistry images for leukocyte infiltration (CD45/DAPI), M2 polarization (CD163/DAPI), M1 polarization (CD86/3,3′‐diaminobenzidine [DAB] staining), and endothelialization (CD31/DAPI), respectively. Scale bar = 100 μm. (iii) CD45, CD163, CD86, and CD31 quantification based on the fluorescent intensity, showing the lowest expression of CD45 and CD86, but also the highest expression of CD163 and CD31. (^#^ *p* < 0.05, ^&&&^ *p* < 0.001, *p < 0.05, ***p < 0.001). Reprinted from Reference 95, Copyright (2021), with permission from the authors. (b) (i) Schematic of a reduced graphene oxide (RGO)‐loaded acellular dermal matrix (ADM) loaded with MSCs tested on a diabetic mouse wound model. (ii) increased collagen (Col) deposition and enhanced neovascularization in the ADM‐RGO‐MSC group compared to control (*p < 0.05, **p < 0.01), showing the greatest wound healing in this group. (iii) Histological images (Masson's trichrome staining) of diabetic wounds after 28 days of treatment, showing significant Col regeneration in all groups. Reprinted with permission from Reference 49. Copyright (2019) American Chemical Society.

For example, Yang et al. investigated the immunomodulatory effect of culturing WJMSCs with various groups, namely Pullulan (Pul)‐Col, Pul‐Col‐AuNPs, Pul‐AuNPs, Pul, and Col, in a Sprague–Dawley rat model; the AuNPs solution was coated on a 15 mm glass coverslip and implanted in the subcutaneous tissue (Figure 4a).^95^ After 4 weeks, CD45 expression (indicative of leukocyte infiltration induced by M1 macrophages) was lowest at ~0.56‐fold in the Pul‐Col‐AuNPs group, followed by ~0.64‐fold in Col‐AuNPs, and ~0.7‐fold in Pul‐AuNPs. CD163 staining showed a ~1.5‐fold increase in expression for the Pul‐Col‐AuNPs group, followed by a ~1.4‐fold increase for the Col‐AuNPs group, and a ~1.3‐fold increase for the Pul‐AuNPs group. M1 macrophages had the lowest expression of CD86 in the Pul‐Col‐AuNPs group (~0.48‐fold decrease), followed by a ~0.5‐fold decrease in Col‐AuNPs and a ~0.62‐fold decrease in Pul‐AuNPs compared to the control, respectively. CD31 (endothelialization capacity) showed the best expression (~1.2‐fold increase) in the Pul‐Col‐AuNPs group, followed by ~1.3‐fold increases in the Col‐AuNPs and Pul‐AuNPs groups compared to the control, respectively. Thus, the authors concluded that the Pul‐Col‐AuNPs nanocomposites demonstrated better biological performance and anti‐immunogenic response.

Fu et al. developed an ICR mouse skin‐derived dermal matrix embedded with reduced graphene oxide nanoparticles (RGONPs) and GFP‐labeled C57BL/6 mouse BMSCs to treat diabetic wounds (Figure 4b).^49^ Briefly, the acellular dermal matrix (ADM) was fabricated by removal of the epidermis using 0.25% Dispase, 0.3% Triton X‐100, and 0.25% trypsin followed by crosslinking with 1‐ethyl‐3‐(3‐(dimethylamino)propyl)carbodiimide hydrochloride (EDC) and *N*‐hydroxysuccinimide. The RGONPs were fabricated by ultrasound exposure of graphene oxide (GO) mixed with l‐(+)‐ascorbic acid and l‐tryptophan at pH 10 with heating and cooling cycles at 80 °C for 24 h intermittently with the ultrasound exposure. The solution was centrifuged and neutralized before allowing it to self‐assemble into nanoparticles on the ADM. Incorporation of the RGONPs increased the scaffold's mechanical properties, including Young's modulus (2.1‐fold), ultimate tensile strength (1.4‐fold), and strain at failure (1.9‐fold). The researchers then tested this scaffold in an in vivo, STZ‐induced diabetic ICR mouse model, demonstrating that the ADM loaded with both RGONPs and MSCs had the greatest improvement in wound healing compared to the nontreated control, ADM + MSC, and ADM + GO + MSC groups. The RGONP‐ and MSC‐laden ADM had demonstrated enhanced vascularization, showing an ~3.2‐, 1.6‐, and 1.3‐fold increase in α‐smooth muscle actin expression at Day 14 and ~2.1‐, 1.3‐, and 1.1‐fold increase in blood vessel diameter at Day 28 compared to the control, ADM + MSC, and ADM + GO + MSC groups, respectively. Similarly, the composite ADM + RGONPs + MSC had an ~1.5‐, 1.4‐, and 1.0‐fold increase in Col deposition after 14 days compared to the control, ADM + MSC, and ADM + GO + MSC groups, respectively.

In another study, Xu et al. fabricated human umbilical cord‐derived mesenchymal stem cell (UCMSC)‐loaded, injectable, thermosensitive chitosan/glycerol phosphate sodium hydrogels embedded with cellulose nanocrystals fabricated by physical crosslinking (mixing in ice bath followed by gelation at 37 °C).^96^ The researchers found decreased TNF‐α and IL‐1β expressions in the inflammatory phase (Days 3 and 7) and increased keratin 1 expression in the remodeling phase (D21) for the cell‐laden composite compared to the PBS‐injected control group, hydrogel alone, and UCMSC alone groups. The enhanced expression of these markers in the cell‐laden composite biomaterial compared with the controls demonstrated superior treatment of full‐thickness cutaneous wounds, evaluated using a specific‐pathogen‐free class SD rat wound model, resulting in a decrease in the inflammation and increase in angiogenesis, cell migration and proliferation, wound closure, Col deposition, re‐epithelialization, and reforming hair follicles.

The incorporation of micron‐sized materials within 3D scaffolds may be used in place of nanomaterials. Westman et al. engineered porcine mesothelium‐derived decellularized ECM into microparticles (MPs).^97^ The MPs were fabricated by passing the decellularized matrix through a knife mill and sieving through 850 and 425 μm screens in order to increase surface area, which in turn can increase cell adhesion. BMSCs were then seeded onto the MPs and the BMSC/MPs were subsequently encapsulated within a fibrin gel. In a nu/nu mouse model, the researchers demonstrated that MSC migration from the MPs into the fibrin gel and wound site occurred within 24 h and increased over time. There was increased cell infiltration, a more developed dermal layer, and greater Col deposition in the MSC‐seeded MP group compared to the control wound that received no treatment, after just 7 days, as observed by Masson's trichrome staining. In a different study by Gonzalez‐Pujana et al., the researchers combined a calcium carbonate slurry, alginate, IFN‐γ‐loaded heparin‐coated agarose beads, and human BMSCs to a solution of Col and polymerized at 37°C to form a cell‐laden hydrogel.^65^ This cell‐laden hydrogel underwent inflammatory licensing overnight with IFN‐γ and TNF‐α, resulting in increase of indoleamine 2,3‐dioxy‐genase 1, galectin‐9, and prostaglandin‐endoperoxide synthase 2/cyclooxygenase 2, indicating an immunosuppressive phenotype in the BMSCs. The authors also demonstrated that the upregulation of these genes were significantly increased for BMSCs in the hydrogel as compared to BMSCs in 2D culture, suggesting that 3D culture and inflammatory licensing work synergistically to enhance the immunomodulatory potential of MSCs.

### 2.2 Synthetic biomaterials

One of the most commonly used synthetic polymers is poly(ethylene glycol) (PEG), a highly hydrophilic, bioinert polymer with a known shielding mechanism that protects biomaterials from eliciting immunogenic responses.^98^ Swartzlander et al. harnessed this potential to develop a C57BL/6 murine MSC‐laden PEG hydrogel capable of combating the foreign body response through both the biomaterial properties and the effects of the MSCs.^52^ In this study the hydrogel was photopolymerized using 0.05% Irgacure I2959 under 365 nm UV light for 10 m. The researchers demonstrated that the MSCs had a major effect on macrophage activation through the secretion of PGE_2_. MSC‐secreted PGE_2_ resulted in macrophage M1‐M2 transition, causing an overall reduction in pro‐inflammatory cytokines; MSCs then sensed the reduced pro‐inflammatory cytokines, resulting in decreased PGE_2_ secretion. PEG further affected this by facilitating this crosstalk via localization of cytokines near encapsulated MSCs, as well as supporting MSC survival.

**FIGURE 5:**
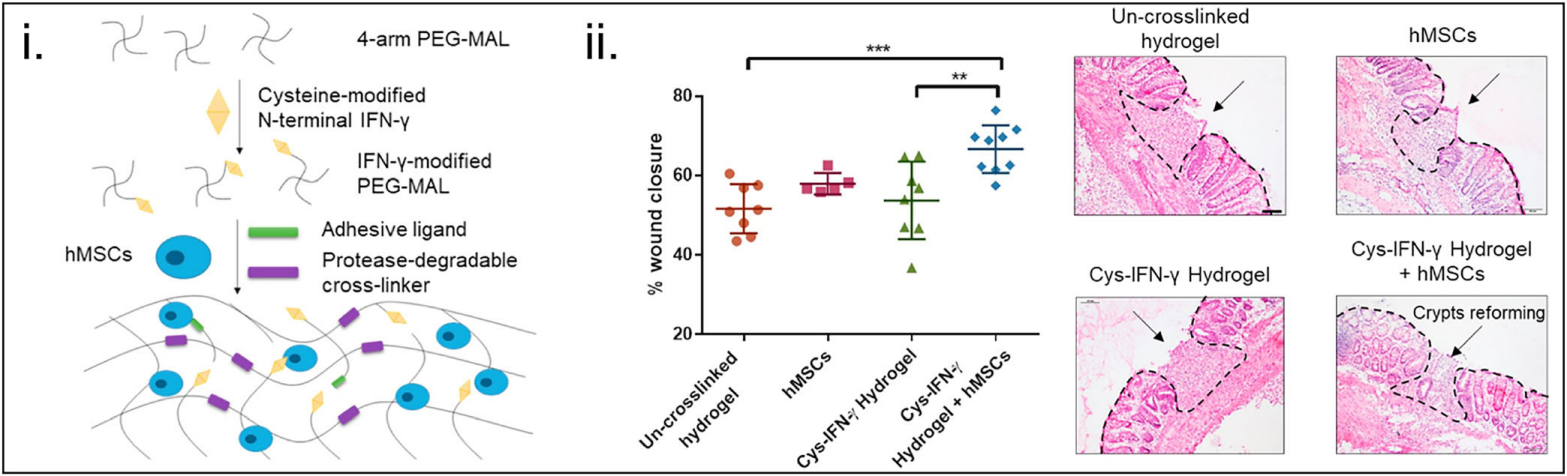
Synthetic polymer‐mesenchymal stem cell (MSC) therapy to enhance wound healing. (i) Schematic depicting cysteine (Cys)‐modified interferon‐gamma (IFN‐γ) tethered to maleimide (mal)‐modified 4‐arm poly(ethylene glycol) (PEG), which is subsequently crosslinked with a protease‐degradable peptide to form a human MSC‐encapsulated bioactive hydrogel. (ii) Wound healing for noncrosslinked hydrogels, MSCs alone, Cys–IFN–γ hydrogels, and Cys–IFN–γ hydrogels encapsulating MSCs after 5 days. Cys–IFN–γ hydrogels encapsulating MSCs had a significant increase in the percentage of wound closure (~20%) compared with the uncrosslinked hydrogel and Cys–IFN–γ hydrogel alone (**p < 0.01, ***p < 0.001). Hematoxylin and eosin staining (H&E) confirmed this morphologically, demonstrating the reformation of crypts in the Cys–IFN–γ hydrogels encapsulating MSCs. Reprinted from Reference 53, Copyright (2019), with permission from Elsevier.

Another reason why PEG is a preferred synthetic polymer is due to its ability to be bio‐functionalized with biochemical signals. For example, Garcia et al. engineered a human MSC‐encapsulated, injectable hydrogel whereby 4‐armed, maleimide‐functionalized PEG was first covalently tethered to cysteine‐modified IFN‐γ, then functionalized with RGD peptide (Figure 5).^53^ The hydrogel was then crosslinked using bi‐cysteine protease‐degradable peptide VPM and dithiothreitol following the functionalization. The effect of this IFN‐γ‐functionalized, MSC‐laden hydrogel on the colonic mucosal wound was examined in vivo in immunocompetent C57/B6 mice. Wound closure for MSC‐encapsulated, IFN–γ‐functionalized hydrogels after 5 days was significantly higher than IFN–γ‐functionalized hydrogel and un‐crosslinked hydrogel control groups by ~1.2‐fold and ~1.3‐fold, respectively. Furthermore, H&E staining showed fewer remaining wounds in the functionalized hydrogel with MSCs compared to the other groups after 5 days.

Promoting cadherin‐engagement has been shown to enhance the MSC secretome.^99^, ^100^ Caldwell et al. demonstrated enhancement via cadherin‐engagement using a porous bio‐click microgel scaffold.^99^ Briefly, the porous bio‐click microgel scaffold was synthesized using an inverse suspension polymerization in hexanes with Span‐90 and Tween‐20 using PEG functionalized with dibenzocylooctyne (PEG‐DBCO) and PEG‐azide (PEG‐N_3_) macromers, and their size was controlled via an applied shear force. RGD, a cellularly adhesive peptide, and GHAVDI(HAVDI), an *N*‐cadherin mimicking peptide, were included in all microgels. BMSCs were encapsulated in microgels with large (190 ± 100 μm), medium (110 ± 60 μm), and small (13 ± 6 μm) diameters. MSCs exhibited greatest clustering in microgels with the large diameter (98% ± 1.6%) compared to 68% ± 19%, and 18% ± 21% for the medium and small diameter microgels, respectively. There were also the largest cluster sizes of MSCs in the microgels with large diameter (40 ± 18 cells/cluster) compared to 7.0 ± 3.0 and 5.0 ± 1.0 cells/cluster for the medium and small diameter microgels, respectively. The broad array of cytokines excreted from MSCs seeded in the microgels, with 48 of the 72 (~60%) detectable cytokines, was also the most elevated in the large diameter scaffold. Large *N*‐cadherin punctae were observed in the large MSC clusters in the microgels with the large diameter; *N*‐cadherins are responsible for adherence junctions between cells. It was then confirmed that the *N*‐cadherin interactions of the large clusters of cells seeded in the large diameter microgel were the reason for the increased cytokine excretion by blocking *N*‐cadherin with a monoclonal antibody against *N*‐cadherin. When *N*‐cadherin was blocked, there was a 10‐fold decrease for 45% of the cytokines in the large diameter microgel condition. When an *N*‐cadherin mimetic peptide, HAVDI, was conjugated to the microgel formulations, the MSC secretary profile was increased in all scaffold conditions. Specifically, of the 80 measured cytokines, 96% of them were increased in the large diameter condition and 86% and 89% were increased in the medium and small diameter conditions, respectively. The increase of MSC cytokine excretion due to *N*‐cadherin interactions also increased the excretion of anti‐inflammatory cytokines and could thus make these cell‐seeded microgel scaffolds a potential method of treatment for chronic nonhealing wounds.

Finally, pluronic is another commonly used synthetic polymer because of its thermosensitivity, allowing pluronic‐based biomaterials to fill irregularly shaped wounds, and its porous structure, allowing for the sustainable release of encapsulated molecules.^101^ Jiao et al. developed WJMSC‐encapsulated Pluronic F‐127 (PF‐127) hydrogels containing sodium ascorbyl phosphate (SAP).^54^ The results of the WJMSC‐loaded system showed expedited wound healing, with approximately 10% of the wound remaining after 2 weeks as opposed to 35% for the PF‐127 + SAP and WJMSCs+SAP groups. Furthermore, the WJMSC‐loaded system promoted regeneration by a 2.1‐ and 1.5‐fold increase in dermis thickness, a reduction of CD86 (M1 macrophages) by 0.38‐ and 0.36‐fold, an increase in CD163 (M2 macrophages) by 2.5‐ and 3‐fold, and an increase of CD31 (angiogenesis) by 2.1‐ and 1.5‐fold, compared to the PF‐127 + SAP and WJMSCs + SAP groups, respectively.

### 2.3 The combination of natural and synthetic biomaterials

Despite their extensive use, naturally occurring polymers remain limited by inadequate mechanical properties.^102^ Alternatively, hydrogels made exclusively from synthetic polymers are often limited by their lack of cell adhesion sites.^103^ Thus, many researchers focus on the combination of natural with synthetic polymers to fabricate hydrogels with the best properties of both: enhanced mechanical properties from the synthetic polymers and bioactivity from natural polymers.

Atallah et al. developed a cell‐instructive, injectable hydrogel by clicking a maleimide‐functionalized heparin GAG to star‐shaped, thiolated PEG using a Michael addition click reaction.^104^ Functionalization of the heparin GAGs with maleimide was achieved by selectively desulfating the heparin GAGs to form different sulfation patterns, which could further modulate the hydrogel's affinity for different cytokines and growth factors, controlling their release and thereby modulating the migration and proliferation of the embedded MSCs. In addition, by changing the physical properties of the hydrogel (e.g., stiffness, swelling degree, and mesh size), the researchers can control cell fate. They found that hydrogels with a building block concentration of 3% w/v exhibited a storage modulus of 1.2 ± 0.07 kPa and a volumetric swelling degree of 1.4 ± 0.2, which was capable of mitigating mechanical irritation of cells. Similarly, Dong et al. also used a Michael addition click reaction to fabricate a PEG‐HA‐RGD hydrogel loaded with ADSCs.^56^ The click reaction allowed the researchers to fabricate hydrogels with tunable mechanical properties and rapid crosslinking (<5 m). They found that the ADSC‐loaded PEG‐HA‐RGD hydrogel was able to treat a second‐degree burn wound in an FVB/NJ murine model. Stereological analysis showed that the hydrogel increased angiogenesis such that the total vascular length was almost 2× greater than that of the hydrogel alone and no treatment control groups. Moreover, this hydrogel decreased the remaining wound area after 11 days to 1.0% ± 0.5% compared to the no treatment control group, which still had 7.6% ± 3.0% wound area remaining, and the hydrogel alone group, which is not significantly different from the no treatment group.

**FIGURE 6:**
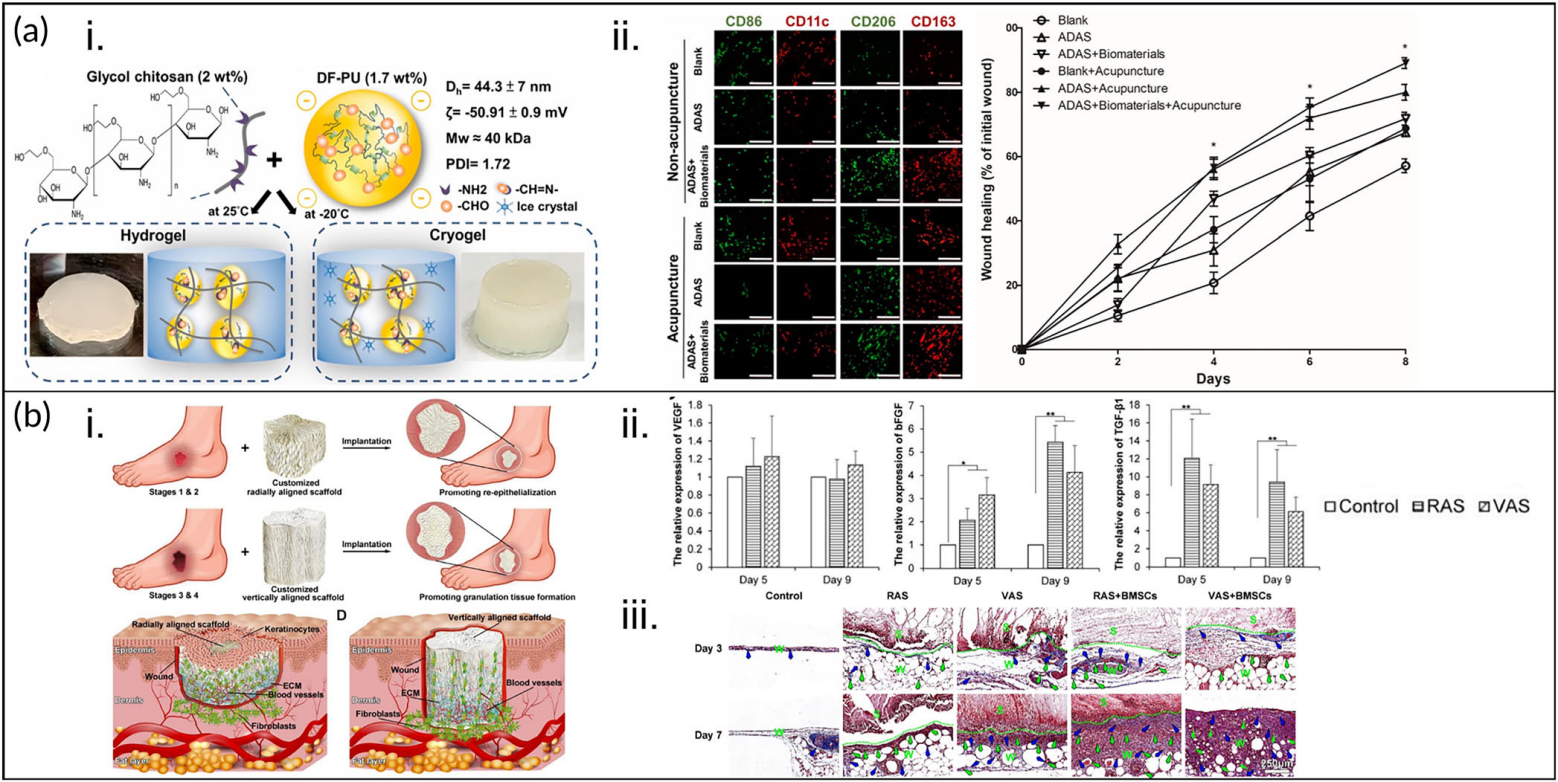
Combination of natural biomaterials with synthetic biomaterials for enhancing chronic wound healing. (a) (i) Illustration of difunctional polyurethane (DF‐PU)‐crosslinked glycol chitosan hydrogel and cryogel formation at 25 °C and −20 °C, respectively. (ii) Immunofluorescent images of CD86 and CD11c (M1 macrophages) and CD206 and CD163 (M2 macrophages), showing that acupuncture effectively reduced the amount of M1‐macrophages present but had no influence on M2‐macrophages for all groups with or without acupuncture. CD86 and CD206 = green, CD11c and CD163 = red. Scale bars = 50 μm. (iii) Wound‐healing rates for different groups from the start of the injury to 8 days, showing the highest healing rate in the ADSC + biomaterials + acupuncture group (*p < 0.05). Reprinted from Reference 55, Copyright (2020), with permission from Elsevier. (b) (i) Illustration of bone marrow‐derived MSC (BMSC)‐loaded radially aligned scaffold (RAS) and vertically aligned scaffold (VAS) to treat superficial and deep diabetic wounds. (ii) The relative expression levels of vascular endothelial growth factor (VEGF), basic fibroblast growth factor (bFGF), and transforming growth factor (TGF)‐β1 after 5 and 9 days, showing increased expression for bFGF and TGF‐β1 compared to the control (*p < 0.05, **p < 0.01). (iii) Masson's trichrome staining on Days 3 and 7 in the control, RAS, VAS, BMSC‐loaded RAS, and BMSC‐loaded VAS groups, showing increased angiogenesis, granulation tissue formation, and collagen (Col) deposition in the scaffold‐treated groups compared with the control group. Green and blue arrows denote new blood vessels and Col fibers, respectively. S and W are the scaffold area and wound area, respectively. Scale bars = 250 μm. Reprinted from Reference 108, Copyright (2020), with permission from Elsevier.

Chen et al. developed a combination of difunctional polyurethane (DF‐PU)‐crosslinked chitosan hydrogel/cryogel loaded with ADSCs, showing improved wound closure in 8 days (Figure 6a).^55^ Briefly, the hydrogel/cryogel was fabricated by a Schiff reaction between amine groups in chitosan and aldehyde groups in DF‐PU. For 2 days, the researchers applied the ADSC‐laden biomaterial in its frozen form due to its potential to absorb wound exudates and remove infectious substances such as pathogenic bacteria in the hemostasis and inflammation phase of wound healing. For the subsequent 6 days, the researchers then used the hydrogel form due to the immunomodulatory properties of DF‐PU in the proliferation and remodeling phase of wound healing. The researchers additionally tested this hydrogel/cryogel in conjunction with acupuncture, which the researchers claimed may have a synergistic effect on chronic wound healing. Using this strategy, the ADSC‐seeded hydrogel/cryogel with acupuncture had the highest rate of wound closure (90.6% ± 3.3%) when compared to the controls, which had wound closure rates of 80% ± 3.5%, 72% ± 2.4%, 72% ± 2.9%, 71% ± 5.8%, 68% ± 1%, 63% ± 2.5%, and 57% ± 3%, respectively, for ADSCs + acupuncture, hydrogel/cryogel + acupuncture, ADSC‐loaded hydrogel/cryogel, acupuncture, ADSCs, hydrogel/cryogel, and blank (PBS) groups. The researchers determined that this improved wound healing was due to the upregulated secretion of stromal cell‐derived factor 1 and TGF‐β1, downregulated expression of TNF‐α and IL‐1β, and activation of C3a and C5a.

While the majority of polymeric biomaterials used in conjunction with MSCs for treating chronic, nonhealing wounds are hydrogels, other forms of polymeric biomaterials have been used as well. In one study, Feldman et al. fabricated an albumin scaffold seeded with MSCs and TGF‐β3 for the treatment of pressure ulcers by mixing a solution made from lyophilized rabbit albumin with disuccinimidyl glutarate‐functionalized PEG.^48^ They found that the MSC‐ and TGF‐β3‐loaded scaffold had a 62% increased healing rate in treating pressure ulcers compared to the no treatment group. Furthermore, the MSC‐ and TGF‐β3‐loaded scaffold showed a 110% increased epithelialization rate (ER) and 45% increased contraction rate (CR) at 7 days post‐surgery. Wounds treated with MSCs had the greatest ER/CR ratio, indicating that the presence of MSCs led to regeneration rather than scarring. In another study, Kraskouski et al. fabricated MSC‐loaded, glutaraldehyde (GA)‐crosslinked pectin/polyvinyl alcohol (PVA) films using solution casting.^50^ By changing the type of pectin (citrus, classic, amid), the molecular weight of the PVA, and the weight ratio of pectin to PVA, the authors were able to affect the mechanical properties of the film, including tensile strength, elongation, elastic modulus, swelling, and stability. For example, by increasing the molecular weight of the PVA from 30 to 145 kDa and maintaining a PVA:pectin weight ratio of 2:1, the tensile strength increased by 79% for amid PVA but decreased by 19% and 5% for citrus and classic PVA, respectively. Further, there was an approximately 2.8‐fold increase, 1.6‐fold decrease, and 1.3‐fold increase in the elongation, Young's modulus, and swelling degree of all types of PVA with an increase in the molecular weight. The researchers also found that the addition of Col and a low amount of GA crosslinker on these pectin/PVA films could improve the adhesion of MSCs to the film's surface by decreasing the stiffness.

Electrospun nanofibers are an attractive wound dressing due to their high surface area to volume ratio, highly porous structure that promotes oxygen and nutrient exchange, capability to absorb wound exudates, mimicry of the native ECM depending on the material chosen, and capability to encapsulate and release drugs and other small molecules.^105^, ^106^ Furthermore, the physical properties of electrospun nanofibers, such as their morphology, porosity, diameter, and alignment have been shown to influence MSC behavior, including proliferation and differentiation,^107^ which makes them excellent candidates as biomaterials for MSC treatment of chronic, nonhealing wounds. Chen et al. fabricated tailored, BMSC‐encapsulated, 3D PF‐127/poly(ε‐caprolactone) nanofiber scaffolds with an aligned fiber structure and diameters of 6, 8, 10, and 12 mm to treat diabetic wounds (Figure 6b).^108^ To investigate the effects of fiber alignment on diabetic wound healing, they designed scaffolds with radially aligned nanofibers or vertically aligned nanofibers and tested these scaffolds on TALLYHO type 2 diabetic mice wound models. Immersing the scaffolds in a 0.5% gelatin solution enhanced their compressive modulus and imparted shape recovery properties. The radially aligned scaffolds (RAS) and vertically aligned scaffolds (VAS) were found to have different contributions to wound healing, with RAS alone having the highest re‐ER (46%± 13%) after 7 days. In addition, while the RAS or VAS alone improved wound‐healing outcomes, loading the scaffolds with BMSCs resulted in synergistic and increased healing. Aligned nanofiber orientation resulted in a ~4.5‐ and ~8‐fold increase in bFGF and TGF‐β1 expression, respectively, after 9 days compared with the control group (BMSCs alone), as well as ~1.2‐fold increased expression of VEGF compared with BMSCs alone after 5 days, which is indicative of increased angiogenesis. As a result, Masson's trichrome staining showed increased Col deposition, which is indicative of wound closure and healing. Furthermore, complete re‐epithelialization was seen for VAS and RAS with and without BMSCs after 10 days but not for the BMSCs alone. Based on these results, the researchers suggested using RAS to enhance re‐epithelialization in superficial wounds and VAS to improve granulation tissue formation in deep wounds. Thus, the researchers were able to develop scaffolds that could be tailored to the degree of diabetic ulcer.

A common concern with the treatment of chronic, nonhealing wounds is their susceptibility to infection.^32^ Silver nanoparticles (AgNPs) have been found to decrease matrix metalloproteinases activity,^109^ exhibit anti‐inflammatory^110^ and anti‐bacterial^111^ properties, and show low cytotoxicity,^4^ which makes them excellent candidates to be used synergistically with MSCs. Mendes et al. designed a two‐layered membrane consisting of a layer of PVA‐AgNPs coated with a layer of Col‐HA fabricated by mixing a 9:1 ratio of Col to HA, freezing at −20°C, lyophilizing, activating with EDC, and exposing to UV radiation; Human ADSCs were seeded in the Col‐HA layer to develop a cutaneous skin substitute.^112^ The researchers carefully selected each component of this biomaterial for its effect on wound healing and skin regeneration: the hydrophilic PVA provides the wound with necessary hydration, and the AgNPs impede microorganism growth on the PVA. The secondary Col‐HA layer interfaces with the MSCs: the Col improves MSC cell adhesion and modification with HA inhibits infections. Finally, the MSCs themselves were chosen due to their immunomodulatory potential. The researchers showed qualitatively that the PVA‐AgNPs/Col‐HA + MSC group exhibited continuous epidermal regeneration and hair follicle growth while the control group (pure PVA) demonstrated granulation tissue and fibrin‐leucocyte crusts.

## 3 CHALLENGES AND FUTURE OUTLOOK

The synergistic potential of MSCs and biomaterials to treat chronic, nonhealing wounds is clear based on the significant recent progress in the literature. However, preclinical progress has not yet translated into clinical progress. Why is this the case? In this section, we discuss the current challenges preventing clinical translation, as well as provide an outlook of next‐generation advances that will pave the way towards clinical use of these therapies.

### 3.1 Clinical trials involving MSCs and biomaterials for chronic, nonhealing wounds

There is no food and drug administration (FDA)‐approved MSC therapy in the US for chronic, nonhealing wounds or other applications. In fact, there is only 1 FDA‐approved stem cell therapy to date, hematopoietic stem cell transplantation, according to the list of approved cellular and gene therapy products on “fda.gov.” Outside of the United States, there are several MSC therapies on the market, including some treatment of chronic, nonhealing wounds, such as Crohn's fistulas, though none appear to incorporate biomaterials to improve the effects of the MSCs.^113^ A number of factors limit the clinical application of MSC therapies, including availability, uncertain number of cells in each dose, low survival, hemocompatibility (safety concern), efficacy, and cost.^114^ Based on the pre‐clinical research conducted thus far, it is clear that biomaterials can help to overcome these limitations.

Currently, there are ~33 clinical trials using MSCs for a variety of chronic, nonhealing wounds at various stages in the clinical trial process (~20 have been completed), according to a search in “clinicaltrials.gov.” Despite the recent literature demonstrating the potential of biomaterials to improve MSC therapies for chronic, nonhealing wounds, only 10 of these clinical trials involve biomaterials (of which only 1 has been completed), thereby highlighting the nascent state of the field and the limited information regarding the success of these therapies clinically. Table 1 provides a list of these biomaterial‐MSC clinical trials, as well as an additional biomaterial‐MSC clinical trial found in the literature. Among these, DFU is the predominant chronic, nonhealing wound condition that is currently being investigated, and the biomaterials being utilized are predominantly naturally derived polymers. In addition, none of these clinical trials utilize decellularized tissue biomaterials or nanomaterials, either alone or in combination with other biomaterials to fabricate advanced composite biomaterial platforms. However, since investigations using synthetic biomaterials, decellularized tissue matrix, nanomaterials, and the combination of natural and synthetic biomaterials are the focus of current preclinical research, we expect that this research will result in the development of the next wave of multi‐functional biomaterial‐MSC clinical trial investigations for chronic, nonhealing wounds.

**TABLE 1:** Clinical trials of MSCs + biomaterials for the treatment of chronic, nonhealing wounds.

Year	Cell type	Biomaterial	Wound type	Trial phase	Clinicaltrial.gov ID or [ref]
2016	UCMSC	Col membrane	DFU	Phase 1/2	NCT02672280
2017	BMSC	Autologous platelet rich gel	DFU	Early Phase 1	NCT03248466
2017	ADSC	Chitosan scaffold	DFU	Phase 1	NCT03259217
2018	BMSC	Col scaffold	DFU	Phase 1	NCT03509870
2018	ADSC	Hydrogel sheet	DFU	Phase 3	NCT03370874
2019	ADSC	Fibrin gel	DFU	Phase 2	NCT03865394
2021	ADSC	Platelet‐rich fibrin	DFU	NA	117
2022	MCAMSC	Heptylamine plasma polymer‐coated silicone	DFU	Phase 1	NCT05165628
2017	ADSC	Col	Venous ulcers	NA	NCT02961699
2015	ADSC	Fibrin sealant (TISSEEL)	Pressure ulcers, DFU	Phase 1	NCT02375802
2010	BMSC	Fibrin	Nonhealing wounds	Phase 1	NCT01751282

Abbreviations: ADSC, adipose‐derived mesenchymal stem cells; BMSC, bone marrow‐derived mesenchymal stem cells; Col, collagen; DFU, diabetic foot ulcer; MCAMSC, mesenchymoangioblast‐derived mesenchymal stem cells; NA, not applicable; UCMSC, umbilical cord‐derived mesenchymal stem cells.

Perhaps an additional existing limitation that affects the transfer of knowledge and the ability of researchers to expand upon previously conducted research is that it remains difficult to directly compare various clinical trials due to differences in dose, time, inclusion and exclusion criteria for selecting patients, wound type, and factors affecting MSC properties, such as MSC source and delivery methods.^15^ More clinical trials, as well as trials that are in later phases, are required in order to have a deeper understanding of the safety and efficacy of MSC‐containing biomaterials for treating chronic, nonhealing wounds. However, the early results from these existing trials support conclusions from preclinical studies in the literature, further demonstrating the potential of these therapies to translate successfully to clinical use and achieve FDA approval.

### 3.2 Advanced composite biomaterials

It is anticipated that continued advances in this field will focus on the fabrication of composite biomaterial platforms. This is due to the enhanced potential of composite biomaterials in terms of their ability to provide tailored signals to the MSCs and wound environments from the scaffold, nanomaterial, and any encapsulated drugs or small molecules, as well as in terms of their ability to mitigate limitations of one biomaterial component by using another biomaterial component. For example, one of the challenges that prevents polymeric wound dressings from clinical translation is off‐target delivery and infection, which can be addressed by adding nanomaterials, antimicrobial agents, sensors, and therapeutic molecules.^9^ In addition, as previously mentioned, combining synthetic polymers with natural polymers can promote scaffold mechanical properties, while simultaneously improving biocompatibility and bioactivity.

The use of nanomaterials within 3D polymeric or decellularized tissue scaffolds has a multitude of advantages, including potential antimicrobial properties (using metallic nanoparticles),^3^, ^7^ controlled and sustained release of encapsulated drugs,^105^ and enhanced physicochemical properties for the biomaterial platform.^49^ However, uncontrolled release of nanoparticles from the scaffolds causing off‐site nanoparticles accumulation,^46^ immunogenic reactions in vivo,^46^ and nanoparticle cytotoxicity remain challenges that must continue to be explored in the literature, particularly as researchers continue to develop new, more advanced nanobiomaterials.^46^

Other challenges associated with advanced, composite biomaterial platforms are their short life span (i.e., rapid degradation, one‐time use), complex fabrication strategies, and the heterogeneity of wounds from patient to patient. Considering these challenges, it is likely that the next generation of advanced composite biomaterials will be designed to be patient‐specific.^3^, ^7^, ^36^, ^116^ This may include manufacturing in situ using advanced techniques (i.e., electrospinning, 3D printing, and microfluidics) such that the biomaterial can adjust to the shape and depth of the wound. It may also include considering the effects of the patient's characteristics (i.e., age, racial background, biological sex, and disease progression) in the biomaterial design.^39^ In addition, there has been some advancement in the fabrication of ‘smart’ composite biomaterials, such as in the development of sensor‐based, cell‐laden nanocomposite hydrogels to show real‐time information about the status of the wound site.^117^

### 3.3 MSC‐derived therapies

In a way, the history of this field of research has followed a certain path: (1) Biomaterials as wound dressings to treat symptoms, (2) MSC therapies to heal wounds, and (3) Synergistic effects of biomaterial‐MSC therapies for enhanced wound healing. Perhaps in an ironic twist, the future of the field seems to be taking a circular approach, with recent literature focusing on the development of MSC‐free therapies. However, these therapies are not the same as the biomaterial wound dressings from the past. Rather than MSC‐free, it is more appropriate to say MSC‐derived. Because of their heterogeneity, complex behavior, and propensity to elicit immunogenic responses or undergo apoptosis during injection, cell‐based therapies are difficult to translate to the clinic.

While combining MSCs and biomaterials can enhance their immunosuppressive capacity, this varies widely due to donor heterogeneity. This was shown by Kwee et al. where they used human BMSCs licensed with or without IFN‐γ and co‐cultured with peripheral blood mononuclear cells from four different donors and compared their varied response on both fibrin and Col I hydrogels normalized to the same weight percentage.^118^ The activity of IDO, an immunosuppressive enzyme, and the expression of PD‐L1, which binds to and inhibits the activation of T‐cells, were strongly upregulated to different magnitudes in the different donors, with IDO activity ranging from an ~22‐fold increase to an ~15‐fold increase, and PD‐L1 expression ranging from an ~38‐fold increase to an ~30‐fold increase, compared to the nearly absent activity in MSCs without licensing on fibrin hydrogels. On Col hydrogels, the IDO activity ranged from an ~20‐fold increase to an ~5‐fold increase, and PD‐L1 expression ranging from an ~63‐fold increase to an ~50‐fold increase compared to MSCs without licensing. This shows great variability in the immunosuppressive functionality of MSCs from different sources, showing some of the difficulties this field currently faces. The authors also found that there were correlations between immunosuppressive capacity and integrin expression, the magnitude of $a_{V}$ and $a_{5}\;$integrin subunit expression on fibrin gels enhanced the immunosuppressive capacity of the MSCs, and on Col gels $\alpha_{2}$ and $\beta_{1}$ integrin subunit expression similarly enhanced the immunosuppressive capacity. This indicated that by sorting for high levels of the integrin subunits for the specific biomaterial to be used, some donor heterogeneity can be limited to give more consistent results.

Recent literature has demonstrated that it is not necessarily the MSCs themselves that are immunomodulatory and cause wound healing, but rather their secretome.^115^ Thus, a recent trend has been to fabricate MSC‐derived biomaterials using components of the MSC secretome for the treatment of chronic, nonhealing wounds.

Exosomes are extracellular vesicles secreted by cells that contain a range of factors, including nucleic acids, proteins, cytokines, growth factors, and more.^119^ MSCs can produce exosomes that contain the immunomodulatory cytokines that the cells secrete,^101^ and researchers have begun to study the influence of these MSC‐derived exosomes within biomaterials for wound healing.^120^ For example, diabetic wound treatment has seen advances by Xiao et al., who incorporated human ADSC‐derived exosomes within a human AM scaffold,^119^ Yang et al., who fabricated a thermosensitive PF‐127 hydrogel loaded with human UCMSC‐derived exosomes,^101^ and Ma et al., who combined UCMSC‐derived small extracellular vesicles with a porcine catecholamine‐modified SIS hydrogel.^121^ Additionally, Shiekh et al. encapsulated ADSC‐derived exosomes and calcium peroxide within an antioxidant polyurethane cryogel,^40^ and Klimczak et al. investigated the effect of a human ADSC secretome‐incorporated Col hydrogel on chronic wound healing in vitro.^122^ Su et al. used MSC exosomes tethered to polyethyleneimine‐modified polycaprolactone fibers (PEFs) seeded with MΦ cell line RAW 264.7 or human umbilical vein endothelial cells to investigate the immune response in excisional skin wounds in a Balb/c female mouse model.^123^ PEF only treatment increased proinflammatory cytokine CD$86^{+}$ expression by 1.6‐fold compared to a no‐treatment control after Day 7. Treatment with exosome‐PEF resulted in an increase in CD$206^{+}$, an immunomodulatory cytokine, by 2‐fold compared to control on Day 7. Exosome‐PEF promoted the presence of regulatory T‐cells by 2‐fold with a higher portion of the T‐cells (18.6% ± 6.4%) secreting IL‐10, an anti‐inflammatory cytokine, compared to the control on Day 7. Due to treatment with exosome‐PEF, there was a remote adaptive immune response as shown in inguinal lymph nodes near skin wounds, where IL‐4 creating CD$86^{+}$ $T_{H}2$ lymphocytes emerged on Day 7 with a 4‐fold increase compared to control. Exosome‐PEF scaffolds were also shown to accelerate wound closure compared to the control (65.1% ± 5.3%), exosomes only (29.2% ± 10.5%), and to a blank PEF scaffold (16.8% ± 7.2%). These results indicate that by combining these PEF scaffolds with MSC derived exosomes, the innate and adaptive immune system can be used to treat skin wounds.

One of the major limitations of exosomes, however, is the complex process to obtain them and the difficulty in controlling their contents.^120^ To simplify the process, other researchers have proposed using lyophilized stem cells or lyophilized stem cell secretomes, suggesting that paracrine factors secreted by the cells can be preserved by lyophilization. For example, Kakabadze et al. fabricated decellularized human AM scaffolds containing lyophilized BMSCs to accelerate the healing rate of radiation burns,^124^ and Ren et al. investigated an ulvan dialdehyde/chitosan/dopamine/AgNPs composite hydrogel loaded with human UCMSCs lyophilized powder (their supernatant after culture in human platelet lysate‐free α‐MEM medium) for the treatment of chronic diabetic wounds.^31^

## 4 CONCLUSIONS

The focus of this review has been on biomaterials‐mediated MSC therapies for the treatment of a variety of chronic, nonhealing wound conditions. As discussed in the introduction, biomaterials treatments (as wound dressings) were the first utilized approach, but the use of biomaterials was primarily for the mitigation of symptoms rather than true healing of the wound. MSC therapies were subsequently investigated for their ability to heal traditionally chronic, nonhealing wounds, demonstrating significant potential in preclinical research and some success in clinical trials. However, their translation to the market has been challenged by limitations surrounding cellular heterogeneity, survival during injection, and complex cellular behavior that is difficult to control, among other factors. Biomaterials can provide support to protect MSCs during injection, as well as provide signals to modulate and control MSC behavior to achieve the desired immunomodulatory behaviors. Thus, the development of MSC‐biomaterial therapies allows researchers to develop advanced treatments for chronic, nonhealing wounds. We will continue to see advances in this field, particularly with composite biomaterial platforms combining natural and synthetic biomaterials, which will ultimately result in their clinical translation.

## AUTHOR CONTRIBUTIONS

**Romina Keshavarz:** Data curation (lead); formal analysis (lead); investigation (lead); visualization (equal); writing – original draft (lead); writing – review and editing (lead). **Sara Olsen:** Data curation (supporting); formal analysis (supporting); writing – review and editing (equal). **Bethany Almeida:** Conceptualization (lead); funding acquisition (lead); project administration (lead); resources (lead); supervision (lead); visualization (equal); writing – original draft (equal); writing – review and editing (equal).

## FUNDING INFORMATION

The authors acknowledge funding support from Clarkson University.

### PEER REVIEW

The peer review history for this article is available at https://www.webofscience.com/api/gateway/wos/peer-review/10.1002/btm2.10598.

## Data Availability

Data sharing is not applicable to this article as no new data were created or analyzed in this study.

## References

[1] Järbrink K , Ni G , Sönnergren H , et al. Prevalence and incidence of chronic wounds and related complications: a protocol for a systematic review. Syst Rev. 2016;5(1):1‐6. 27609108 10.1186/s13643-016-0329-y PMC5017042

[2] Han G , Ceilley R . Chronic wound healing: a review of current management and treatments. Adv Ther. 2017;34(3):599‐610. 28108895 10.1007/s12325-017-0478-y PMC5350204

[3] Opt Veld RC , Walboomers XF , Jansen JA , Wagener FADTG . Design considerations for hydrogel wound dressings: strategic and molecular. Advances. 2020;26(3):230‐248. 10.1089/ten.TEB.2019.0281 31928151

[4] Nethi SK , Das S , Patra CR , Mukherjee S . Recent advances in inorganic nanomaterials for wound‐healing applications. Biomater Sci. 2019;7(7):2652‐2674. 31094374 10.1039/c9bm00423h

[5] Kuehlmann B , Bonham CA , Zucal I , Prantl L , Gurtner GC . Mechanotransduction in wound healing and fibrosis. J Clin Med. 2020;9(5):1423. 32403382 10.3390/jcm9051423 PMC7290354

[6] Sen CK . Human wounds and its burden: an updated compendium of estimates. Adv Wound Care (New Rochelle). 2019;8(2):39‐48. 30809421 10.1089/wound.2019.0946 PMC6389759

[7] Przekora A . A concise review on tissue engineered artificial skin grafts for chronic wound treatment: can we reconstruct functional skin tissue In vitro? Cells. 2020;9(7):1622. 32640572 10.3390/cells9071622 PMC7407512

[8] Bardill JR , Laughter MR , Stager M , Liechty KW , Krebs MD , Zgheib C . Topical gel‐based biomaterials for the treatment of diabetic foot ulcers. Acta Biomater. 2022;138:73‐91. 34728428 10.1016/j.actbio.2021.10.045 PMC8738150

[9] Maaz Arif M , Khan SM , Gull N , et al. Polymer‐based biomaterials for chronic wound management: promises and challenges. Int J Pharm. 2021;598:120270. 33486030 10.1016/j.ijpharm.2021.120270

[10] Zawani M , Fauzi MB . Injectable hydrogels for chronic skin wound management: a concise review. Biomedicines. 2021;9(5):527. 34068490 10.3390/biomedicines9050527 PMC8150772

[11] Ramirez‐Acuña JM , Cardenas‐Cadena SA , Marquez‐Salas PA , et al. Diabetic foot ulcers: current advances in antimicrobial therapies and emerging treatments. Antibiotics. 2019;8(4):193. 31652990 10.3390/antibiotics8040193 PMC6963879

[12] Chang C , Yan J , Yao Z , Zhang C , Li X , Mao HQ . Effects of mesenchymal stem cell‐derived paracrine signals and their delivery strategies. Adv Healthc Mater. 2021;10(7):2001689. 33433956 10.1002/adhm.202001689 PMC7995150

[13] da Silva ML , Fontes AM , Covas DT , Caplan AI . Mechanisms involved in the therapeutic properties of mesenchymal stem cells. Cytokine Growth Factor Rev. 2009;20(5‐6):419‐427. 19926330 10.1016/j.cytogfr.2009.10.002

[14] Krasilnikova OA , Baranovskii DS , Lyundup AV , Shegay PV , Kaprin AD , Klabukov ID . Stem and somatic cell monotherapy for the treatment of diabetic foot ulcers: review of clinical studies and mechanisms of action. Stem Cell Rev Rep. 2022;18(6):1974‐1985. 35476187 10.1007/s12015-022-10379-z

[15] Huang YZ , Gou M , Da LC , Zhang WQ , Xie HQ . Mesenchymal stem cells for chronic wound healing: current status of preclinical and clinical. Studies. 2020;26(6):555‐570. 10.1089/ten.TEB.2019.0351 32242479

[16] Shojaei F , Rahmati S , Banitalebi DM . A review on different methods to increase the efficiency of mesenchymal stem cell‐based wound therapy. Wound Repair Regen. 2019;27(6):661‐671. 31298446 10.1111/wrr.12749

[17] Lee DE , Ayoub N , Agrawal DK . Mesenchymal stem cells and cutaneous wound healing: novel methods to increase cell delivery and therapeutic efficacy. Stem Cell Res Ther. 2016;7(1):1‐8. 26960535 10.1186/s13287-016-0303-6 PMC4784457

[18] Las HK , Igartua M , Santos‐Vizcaino E , Hernandez RM . Cell‐based dressings: a journey through chronic wound management. Biomater Adv. 2022;135:212738. 35929212 10.1016/j.bioadv.2022.212738

[19] Song N , Scholtemeijer M , Shah K . Mesenchymal stem cell immunomodulation: mechanisms and therapeutic potential. Trends Pharmacol Sci. 2020;41(9):653‐664. 32709406 10.1016/j.tips.2020.06.009 PMC7751844

[20] Wang M , Yuan Q , Xie L . Mesenchymal stem cell‐based immunomodulation: properties and clinical application. Stem Cells Int. 2018;2018:3057624. 30013600 10.1155/2018/3057624 PMC6022321

[21] Riha SM , Maarof M , Fauzi MB . Synergistic effect of biomaterial and stem cell for skin tissue engineering in cutaneous wound healing: a concise review. Polymers. 2021;13(10):1546. 34065898 10.3390/polym13101546 PMC8150744

[22] Thakur G , Bok EY , Kim SB , et al. Scaffold‐free 3D culturing enhance pluripotency, immunomodulatory factors, and differentiation potential of Wharton's jelly‐mesenchymal stem cells. Eur J Cell Biol. 2022;101(3):151245. 35667339 10.1016/j.ejcb.2022.151245

[23] Murphy KC , Whitehead J , Zhou D , Ho SS , Leach JK . Engineering fibrin hydrogels to promote the wound healing potential of mesenchymal stem cell spheroids. Acta Biomater. 2017;64:176‐186. 28987783 10.1016/j.actbio.2017.10.007 PMC5682213

[24] Madl CM , Heilshorn SC , Blau HM . Bioengineering strategies to accelerate stem cell therapeutics. Nature. 2018;557(7705):335‐342. 29769665 10.1038/s41586-018-0089-z PMC6773426

[25] Yang Z , Concannon J , Ng KS , et al. Tetrandrine identified in a small molecule screen to activate mesenchymal stem cells for enhanced immunomodulation. Scientific Rep. 2016;6(1):1‐10. 10.1038/srep30263 PMC4960598 27457881

[26] Saldaña L , Bensiamar F , Vallés G , Mancebo FJ , García‐Rey E , Vilaboa N . Immunoregulatory potential of mesenchymal stem cells following activation by macrophage‐derived soluble factors. Stem Cell Res Ther. 2019;10(1):1‐15. 30760316 10.1186/s13287-019-1156-6 PMC6375172

[27] Qian Z , Wang H , Bai Y , et al. Improving chronic diabetic wound healing through an injectable and self‐healing hydrogel with platelet‐rich plasma release. ACS Appl Mater Interfaces. 2020;12(50):55659‐55674. 33327053 10.1021/acsami.0c17142

[28] Zhao X , Li Q , Guo Z , Li Z . Constructing a cell microenvironment with biomaterial scaffolds for stem cell therapy. Stem Cell Res Ther. 2021;12(1):1‐13. 34809719 10.1186/s13287-021-02650-w PMC8607654

[29] Mitrousis N , Fokina A , Shoichet MS . Biomaterials for cell transplantation. Nat Rev Mater. 2018;3(11):441‐456.

[30] Yang R , Liu X , Ren Y , et al. Injectable adaptive self‐healing hyaluronic acid/poly (γ‐glutamic acid) hydrogel for cutaneous wound healing. Acta Biomater. 2021;127:102‐115. 33813093 10.1016/j.actbio.2021.03.057

[31] Ren Y , Ailierken A , Zhao L , et al. hUC‐MSCs lyophilized powder loaded polysaccharide ulvan driven functional hydrogel for chronic diabetic wound healing. Carbohydr Polym. 2022;288:119404. 35450656 10.1016/j.carbpol.2022.119404

[32] Kharaziha M , Baidya A , Annabi N , Kharaziha M , Baidya A , Annabi N . Rational Design of Immunomodulatory Hydrogels for chronic wound healing. Adv Mater. 2021;33(39):2100176. 10.1002/adma.202100176 PMC8489436 34251690

[33] Li J , Liu Y , Zhang Y , et al. Biophysical and biochemical cues of biomaterials guide mesenchymal stem cell behaviors. Front Cell Dev Biol. 2021;9:397. 10.3389/fcell.2021.640388 PMC8027358 33842464

[34] Chen Y , Shu Z , Qian K , Wang J , Zhu H . Harnessing the properties of biomaterial to enhance the immunomodulation of mesenchymal. Stem Cells. 2019;25(6):492‐499. 10.1089/ten.TEB.2019.0131 31436142

[35] Abbasi A , Imaichi S , Ling V , Shukla A . Mesenchymal stem cell behavior on soft hydrogels with aligned surface topographies. ACS Appl Bio Mater. 2022;5:1890‐1900. 10.1021/acsabm.1c01260 35199983

[36] Castaño O , Pérez‐Amodio S , Navarro‐Requena C , Mateos‐Timoneda MÁ , Engel E . Instructive microenvironments in skin wound healing: biomaterials as signal releasing platforms. Adv Drug Deliv Rev. 2018;129:95‐117. 29627369 10.1016/j.addr.2018.03.012

[37] Wang S , Hashemi S , Stratton S , Arinzeh TL . The effect of physical cues of biomaterial scaffolds on stem cell behavior. Adv Healthc Mater. 2021;10(3):2001244. 10.1002/adhm.202001244 33274860

[38] Vining KH , Stafford A , Mooney DJ . Sequential modes of crosslinking tune viscoelasticity of cell‐instructive hydrogels. Biomaterials. 2019;188:187‐197. 30366219 10.1016/j.biomaterials.2018.10.013 PMC6279497

[39] Julier Z , Park AJ , Briquez PS , Martino MM . Promoting tissue regeneration by modulating the immune system. Acta Biomater. 2017;53:13‐28. 28119112 10.1016/j.actbio.2017.01.056

[40] Shiekh PA , Singh A , Kumar A . Exosome laden oxygen releasing antioxidant and antibacterial cryogel wound dressing OxOBand alleviate diabetic and infectious wound healing. Biomaterials. 2020;249:120020. 32305816 10.1016/j.biomaterials.2020.120020

[41] Negut I , Dorcioman G , Grumezescu V . Scaffolds for wound healing applications. Polymers. 2020;12(9):2010. 32899245 10.3390/polym12092010 PMC7563417

[42] Liu J , Shi R , Hua Y , Gao J , Chen Q , Xu L . A new cyanoacrylate‐poly(lactic acid)‐based system for a wound dressing with on‐demand removal. Mater Lett. 2021;293:129666.

[43] Zhang Y , Luo J , Zhang Q , Deng T . Growth factors, as biological macromolecules in bioactivity enhancing of electrospun wound dressings for diabetic wound healing: a review. Int J Biol Macromol. 2021;193:205‐218. 34627847 10.1016/j.ijbiomac.2021.09.210

[44] Zhou S , Wang Q , Huang A , Fan H , Yan S , Zhang Q . Advances in skin wound and scar repair by polymer scaffolds. Molecules. 2021;26(20):6110. 34684690 10.3390/molecules26206110 PMC8541489

[45] Zhong Y , Xiao H , Seidi F , Jin Y . Natural polymer‐based antimicrobial hydrogels without synthetic antibiotics as wound dressings. Biomacromolecules. 2020;21(8):2983‐3006. 32672446 10.1021/acs.biomac.0c00760

[46] Blanco‐Fernandez B , Castaño O , Mateos‐Timoneda MÁ , Engel E , Pérez‐Amodio S . Nanotechnology approaches in chronic wound healing. Adv Wound Care (New Rochelle). 2021;10(5):234‐256. 32320364 10.1089/wound.2019.1094 PMC8035922

[47] Kong F , Mehwish N , Lee BH . Emerging albumin hydrogels as personalized biomaterials. Acta Biomater. 2023;157:67‐90. 36509399 10.1016/j.actbio.2022.11.058

[48] Feldman DS , McCauley JF . Mesenchymal stem cells and transforming growth factor‐β3 (TGF‐β3) to enhance the regenerative ability of an albumin scaffold in full thickness wound healing. J Function Biomater. 2018;9(4):65. 10.3390/jfb9040065 PMC6306712 30441760

[49] Fu J , Zhang Y , Chu J , et al. Reduced graphene oxide incorporated acellular dermal composite scaffold enables efficient local delivery of mesenchymal stem cells for accelerating diabetic wound healing. ACS Biomater Sci Eng. 2019;5(8):4054‐4066. 33448807 10.1021/acsbiomaterials.9b00485

[50] Kraskouski A , Hileuskaya K , Kulikouskaya V , et al. Polyvinyl alcohol and pectin blended films: preparation, characterization, and mesenchymal stem cells attachment. J Biomed Mater Res A. 2021;109(8):1379‐1392. 33252172 10.1002/jbm.a.37130

[51] Zhao L , Guo Z , Chen K , et al. Combined transplantation of mesenchymal stem cells and endothelial Colony‐forming cells accelerates refractory diabetic foot ulcer healing. Stem Cells Int. 2020;2020:8863649. 33061991 10.1155/2020/8863649 PMC7545465

[52] Swartzlander MD , Blakney AK , Amer LD , Hankenson KD , Kyriakides TR , Bryant SJ . Immunomodulation by mesenchymal stem cells combats the foreign body response to cell‐laden synthetic hydrogels. Biomaterials. 2015;41:79‐88. 25522967 10.1016/j.biomaterials.2014.11.020 PMC5577802

[53] García JR , Quirós M , Han WM , et al. IFN‐γ‐tethered hydrogels enhance mesenchymal stem cell‐based immunomodulation and promote tissue repair. Biomaterials. 2019;220:119403. 31401468 10.1016/j.biomaterials.2019.119403 PMC6717550

[54] Jiao Y , Chen X , Niu Y , et al. Wharton's jelly mesenchymal stem cells embedded in PF‐127 hydrogel plus sodium ascorbyl phosphate combination promote diabetic wound healing in type 2 diabetic rat. Stem Cell Res Ther. 2021;12(1):1‐15. 34717751 10.1186/s13287-021-02626-w PMC8557497

[55] Chen TY , Wen TK , Dai NT , Hsu SH . Cryogel/hydrogel biomaterials and acupuncture combined to promote diabetic skin wound healing through immunomodulation. Biomaterials. 2021;269:120608. 33388690 10.1016/j.biomaterials.2020.120608

[56] Dong Y , Cui M , Qu J , et al. Conformable hyaluronic acid hydrogel delivers adipose‐derived stem cells and promotes regeneration of burn injury. Acta Biomater. 2020;108:56‐66. 32251786 10.1016/j.actbio.2020.03.040

[57] Yang M , He S , Su Z , Yang Z , Liang X , Wu Y . Thermosensitive injectable chitosan/collagen/β‐glycerophosphate composite hydrogels for enhancing wound healing by encapsulating mesenchymal stem cell spheroids. ACS Omega. 2020;5(33):21015‐21023. 32875238 10.1021/acsomega.0c02580 PMC7450604

[58] Otero G , Agorio C , Sujanov A , et al. Autologous bone marrow–derived cells for venous leg ulcers treatment: a pilot study. Cytotherapy. 2019;21(2):189‐199. 30700393 10.1016/j.jcyt.2019.01.002

[59] Fahimirad S , Ajalloueian F . Naturally‐derived electrospun wound dressings for target delivery of bio‐active agents. Int J Pharm. 2019;566:307‐328. 31125714 10.1016/j.ijpharm.2019.05.053

[60] Divyashri G , Badhe RV , Sadanandan B , et al. Applications of hydrogel‐based delivery systems in wound care and treatment: an up‐to‐date review. Polym Adv Technol. 2022;33(7):2025‐2043.

[61] Bernhard JC , Vunjak‐Novakovic G . Should we use cells, biomaterials, or tissue engineering for cartilage regeneration? Stem Cell Res Ther. 2016;7(1):1‐9. 27089917 10.1186/s13287-016-0314-3 PMC4836146

[62] Park S , Park KM . Engineered polymeric hydrogels for 3D tissue models. Polymers. 2016;8(1):23. 30979118 10.3390/polym8010023 PMC6432530

[63] Liu H , Wang C , Li C , et al. A functional chitosan‐based hydrogel as a wound dressing and drug delivery system in the treatment of wound healing. RSC Adv. 2018;8(14):7533‐7549. 35539132 10.1039/c7ra13510f PMC9078458

[64] Wei W , Zhang Q , Zhou W , et al. Immunomodulatory application of engineered hydrogels in regenerative medicine. Appl Mater Today. 2019;14:126‐136.

[65] Gonzalez‐Pujana A , Vining KH , Zhang DKY , et al. Multifunctional biomimetic hydrogel systems to boost the immunomodulatory potential of mesenchymal stromal cells. Biomaterials. 2020;257:120266. 32763614 10.1016/j.biomaterials.2020.120266 PMC7477339

[66] Guo J , Hu H , Gorecka J , et al. Adipose‐derived mesenchymal stem cells accelerate diabetic wound healing in a similar fashion as bone marrow‐derived cells. Am J Physiol Cell Physiol. 2018;315(6):C885‐C896. 30404559 10.1152/ajpcell.00120.2018 PMC6336941

[67] Gómez‐Aristizábal A , Kim KP , Viswanathan S . A systematic study of the effect of different molecular weights of hyaluronic acid on mesenchymal stromal cell‐mediated immunomodulation. PloS One. 2016;11(1):e0147868. 26820314 10.1371/journal.pone.0147868 PMC4731468

[68] Lu TY , Yu KF , Kuo SH , Cheng NC , Chuang EY , Yu JS . Enzyme‐crosslinked gelatin hydrogel with adipose‐derived stem cell spheroid facilitating wound repair in the murine burn model. Polymers. 2020;12(12):2997. 33339100 10.3390/polym12122997 PMC7765510

[69] Liu Y , Liu Y , Wu M , et al. Adipose‐derived mesenchymal stem cell‐loaded β‐chitin nanofiber hydrogel promote wound healing in rats. J Mater Sci Mater Med. 2022;33(2):1‐14. 10.1007/s10856-021-06630-7 PMC8776676 35050422

[70] Bai H , Kyu‐Cheol N , Wang Z , et al. Regulation of inflammatory microenvironment using a self‐healing hydrogel loaded with BM‐MSCs for advanced wound healing in rat diabetic foot ulcers. J Tissue Eng. 2020;11. 10.1177/2041731420947242 PMC7444096 32913623

[71] Hsu SH , Hsieh PS . Self‐assembled adult adipose‐derived stem cell spheroids combined with biomaterials promote wound healing in a rat skin repair model. Wound Repair Regen. 2015;23(1):57‐64. 25421559 10.1111/wrr.12239

[72] Ren J , Chi J , Wang B , et al. Three‐dimensional cultivation of human adipose‐derived stem cells with human decellularized adipose tissue matrix scaffold promotes diabetic wound healing. Colloids Surf A Physicochem Eng Asp. 2022;640:128478.

[73] Chou PR , Lin YN , Wu SH , et al. Supercritical carbon dioxide‐decellularized porcine acellular dermal matrix combined with autologous adipose‐derived stem cells: its role in accelerated diabetic wound healing. Int J Med Sci. 2020;17(3):354‐367. 32132871 10.7150/ijms.41155 PMC7053351

[74] Somaiah C , Kumar A , Mawrie D , et al. Collagen promotes higher adhesion, survival and proliferation of mesenchymal stem cells. PloS One. 2015;10(12):e0145068. 26661657 10.1371/journal.pone.0145068 PMC4678765

[75] Xu H , Wang J , Wu D , Qin D . A hybrid hydrogel encapsulating human umbilical cord mesenchymal stem cells enhances diabetic wound healing. J Mater Sci Mater Med. 2022;33(8):1‐14. 10.1007/s10856-022-06681-4 PMC9293866 35849219

[76] Murphy KC , Hughbanks ML , Binder BYK , Vissers CB , Leach JK . Engineered fibrin gels for parallel stimulation of mesenchymal stem cell proangiogenic and osteogenic potential. Ann Biomed Eng. 2015;43(8):2010‐2021. 25527322 10.1007/s10439-014-1227-x PMC4475511

[77] Long L , Hu C , Liu W , et al. Injectable multifunctional hyaluronic acid/methylcellulose hydrogels for chronic wounds repairing. Carbohydr Polym. 2022;289:119456. 35483858 10.1016/j.carbpol.2022.119456

[78] Hu H , Ye B , Lv Y , Zhang Q . Preparing antibacterial and in‐situ formable double crosslinking chitosan/hyaluronan composite hydrogels. Mater Lett. 2019;254:17‐20.

[79] Shukla A , Choudhury S , Chaudhary G , et al. Chitosan and gelatin biopolymer supplemented with mesenchymal stem cells (Velgraft®) enhanced wound healing in goats (Capra hircus): involvement of VEGF, TGF and CD31. J Tissue Viability. 2021;30(1):59‐66. 33386237 10.1016/j.jtv.2020.12.002

[80] Zhang X , Chen X , Hong H , Hu R , Liu J , Liu C . Decellularized extracellular matrix scaffolds: recent trends and emerging strategies in tissue engineering. Bioact Mater. 2022;10:15‐31. 34901526 10.1016/j.bioactmat.2021.09.014 PMC8637010

[81] Solarte David VA , Güiza‐Argüello VR , Arango‐Rodríguez ML , Sossa CL , Becerra‐Bayona SM . Decellularized tissues for wound healing: towards closing the gap between scaffold design and effective extracellular matrix remodeling. Front Bioeng Biotechnol. 2022;10:194. 10.3389/fbioe.2022.821852 PMC8896438 35252131

[82] Hu C , Liu W , Long L , et al. Microenvironment‐responsive multifunctional hydrogels with spatiotemporal sequential release of tailored recombinant human collagen type III for the rapid repair of infected chronic diabetic wounds. J Mater Chem B. 2021;9(47):9684‐9699. 34821252 10.1039/d1tb02170b

[83] Nguyen MTN , Tran HLB . Fabrication of an injectable acellular adipose matrix for soft tissue regeneration. Journal of Science: Advanced Materials and Devices. 2021;6(1):1‐10.

[84] Chen Z , Zhang B , Shu J , et al. Human decellularized adipose matrix derived hydrogel assists mesenchymal stem cells delivery and accelerates chronic wound healing. J Biomed Mater Res A. 2021;109(8):1418‐1428. 33253453 10.1002/jbm.a.37133

[85] Elkhenany H , El‐Derby A , Abd Elkodous M , Salah RA , Lotfy A , El‐Badri N . Applications of the amniotic membrane in tissue engineering and regeneration: the hundred‐year challenge. Stem Cell Res Ther. 2022;13(1):1‐19. 35012669 10.1186/s13287-021-02684-0 PMC8744057

[86] Hashemi SS , Mohammadi AA , Moshirabadi K , Zardosht M . Effect of dermal fibroblasts and mesenchymal stem cells seeded on an amniotic membrane scaffold in skin regeneration: a case series. J Cosmet Dermatol. 2021;20(12):4040‐4047. 33656768 10.1111/jocd.14043

[87] Wang CH , Hsieh DJ , Periasamy S , et al. Regenerative porcine dermal collagen matrix developed by supercritical carbon dioxide extraction technology: role in accelerated wound healing. Materialia (Oxf). 2020;9:100576.

[88] Jiang YL , Le WZ , Fan ZX , et al. Human adipose‐derived stem cell‐loaded small intestinal submucosa as a bioactive wound dressing for the treatment of diabetic wounds in rats. Biomaterials Advances. 2022;136:212793. 35929325 10.1016/j.bioadv.2022.212793

[89] Chu J , Shi P , Deng X , et al. Dynamic multiphoton imaging of acellular dermal matrix scaffolds seeded with mesenchymal stem cells in diabetic wound healing. J Biophotonics. 2018;11(7):e201700336. 29575792 10.1002/jbio.201700336

[90] Rashtbar M , Hadjati J , Ai J , et al. Critical‐sized full‐thickness skin defect regeneration using ovine small intestinal submucosa with or without mesenchymal stem cells in rat model. J Biomed Mater Res B Appl Biomater. 2018;106(6):2177‐2190. 29052357 10.1002/jbm.b.34019

[91] Bo Q , Yan L , Li H , et al. Decellularized dermal matrix‐based photo‐crosslinking hydrogels as a platform for delivery of adipose derived stem cells to accelerate cutaneous wound healing. Mater des. 2020;196:109152.

[92] Sun L , Zhao Y , Dong C , et al. Nanoparticle optical properties: size dependence of a single gold spherical nanoparticle. J Phys Conf Ser. 2018;1083(1):012040.

[93] Van de Walle A , Perez JE , Abou‐Hassan A , Hémadi M , Luciani N , Wilhelm C . Magnetic nanoparticles in regenerative medicine: what of their fate and impact in stem cells? Mater Today Nano. 2020;11:100084.

[94] Zhang L , Gu FX , Chan JM , Wang AZ , Langer RS , Farokhzad OC . Nanoparticles in medicine: therapeutic applications and developments. Clin Pharmacol Ther. 2008;83(5):761‐769. 17957183 10.1038/sj.clpt.6100400

[95] Yang MY , Liu BS , Huang HY , et al. Engineered pullulan‐collagen‐gold nano composite improves mesenchymal stem cells neural differentiation and inflammatory regulation. Cell. 2021;10(12):3276. 10.3390/cells10123276 PMC8699622 34943784

[96] Xu H , Huang S , Wang J , et al. Enhanced cutaneous wound healing by functional injectable thermo‐sensitive chitosan‐based hydrogel encapsulated human umbilical cord‐mesenchymal stem cells. Int J Biol Macromol. 2019;137:433‐441. 31271797 10.1016/j.ijbiomac.2019.06.246

[97] Westman AM , Goldstein RL , Bradica G , et al. Decellularized extracellular matrix microparticles seeded with bone marrow mesenchymal stromal cells for the treatment of full‐thickness cutaneous wounds. J Biomater Appl. 2019;33(8):1070‐1079. 30651054 10.1177/0885328218824759

[98] Adel IM , Elmeligy MF , Elkasabgy NA . Conventional and recent trends of scaffolds fabrication: a superior mode for tissue engineering. Pharmaceutics. 2022;14(2):306. 35214038 10.3390/pharmaceutics14020306 PMC8877304

[99] Caldwell AS , Rao VV , Golden AC , Anseth KS . Porous bio‐click microgel scaffolds control hMSC interactions and promote their secretory properties. Biomaterials. 2020;232:119725. 31918222 10.1016/j.biomaterials.2019.119725 PMC7047645

[100] Qazi TH , Mooney DJ , Duda GN , Geissler S . Niche‐mimicking interactions in peptide‐functionalized 3D hydrogels amplify mesenchymal stromal cell paracrine effects. Biomaterials. 2020;230:119639. 31776021 10.1016/j.biomaterials.2019.119639

[101] Yang J , Chen Z , Pan D , Li H , Shen J . Umbilical cord‐derived mesenchymal stem cell‐derived exosomes combined pluronic F127 hydrogel promote chronic diabetic wound healing and complete skin regeneration. Int J Nanomedicine. 2020;15:5911‐5926. 32848396 10.2147/IJN.S249129 PMC7429232

[102] Juncos Bombin AD , Dunne NJ , McCarthy HO . Electrospinning of natural polymers for the production of nanofibres for wound healing applications. Mater Sci Eng C. 2020;114:110994. 10.1016/j.msec.2020.110994 32993991

[103] Suamte L , Tirkey A , Babu PJ . Design of 3D smart scaffolds using natural, synthetic and hybrid derived polymers for skin regenerative applications. Smart Mater Med. 2023;4:243‐256.

[104] Atallah P , Schirmer L , Tsurkan M , et al. In situ‐forming, cell‐instructive hydrogels based on glycosaminoglycans with varied sulfation patterns. Biomaterials. 2018;181:227‐239. 30092371 10.1016/j.biomaterials.2018.07.056

[105] Kamoun EA , Loutfy SA , Hussein Y , Kenawy ERS . Recent advances in PVA‐polysaccharide based hydrogels and electrospun nanofibers in biomedical applications: a review. Int J Biol Macromol. 2021;187:755‐768. 34358597 10.1016/j.ijbiomac.2021.08.002

[106] Li T , Sun M , Wu S . State‐of‐the‐art review of electrospun gelatin‐based nanofiber dressings for wound healing applications. Nanomaterials. 2022;12(5):784. 35269272 10.3390/nano12050784 PMC8911957

[107] Gizaw M , Faglie A , Pieper M , Poudel S , Chou SF . The role of electrospun fiber scaffolds in stem cell therapy for skin tissue regeneration. Med One. 2019;4:e190002. 30972372 10.20900/mo.20190002 PMC6453140

[108] Chen S , Wang H , Su Y , et al. Mesenchymal stem cell‐laden, personalized 3D scaffolds with controlled structure and fiber alignment promote diabetic wound healing. Acta Biomater. 2020;108:153‐167. 32268240 10.1016/j.actbio.2020.03.035 PMC7207021

[109] Wright JB , Lam K , Buret AG , Olson ME , Burrell RE . Early healing events in a porcine model of contaminated wounds: effects of nanocrystalline silver on matrix metalloproteinases, cell apoptosis, and healing. Wound Repair Regen. 2002;10(3):141‐151. 12100375 10.1046/j.1524-475x.2002.10308.x

[110] Kalantari K , Mostafavi E , Afifi AM , et al. Wound dressings functionalized with silver nanoparticles: promises and pitfalls. Nanoscale. 2020;12(4):2268‐2291. 31942896 10.1039/c9nr08234d

[111] Masood N , Ahmed R , Tariq M , et al. Silver nanoparticle impregnated chitosan‐PEG hydrogel enhances wound healing in diabetes induced rabbits. Int J Pharm. 2019;559:23‐36. 30668991 10.1016/j.ijpharm.2019.01.019

[112] Mendes D , Hausen MA , Asami J , et al. A new dermal substitute containing polyvinyl alcohol with silver nanoparticles and collagen with hyaluronic acid: In vitro and In vivo approaches. Antibiotics. 2021;10(6):742. 34205394 10.3390/antibiotics10060742 PMC8235042

[113] Levy O , Kuai R , Siren EMJ , et al. Shattering barriers toward clinically meaningful MSC therapies. Sci Adv. 2020;6(30):eaba6884. 32832666 10.1126/sciadv.aba6884 PMC7439491

[114] Wechsler ME , Rao VV , Borelli AN , Anseth KS . Engineering the MSC Secretome: a hydrogel focused approach. Adv Healthc Mater. 2021;10(7):2001948. 10.1002/adhm.202001948 PMC8035320 33594836

[115] Khalil C , Chaker D , Salameh R , et al. Autologous adipose‐derived mesenchymal stem cells embedded in platelet‐rich fibrin in diabetic foot ulcers. Open J Regenerative Med. 2021;10(2):19‐30.

[116] Savoji H , Godau B , Hassani MS , Akbari M . Skin tissue substitutes and biomaterial risk assessment and testing. Front Bioeng Biotechnol. 2018;6:86. 30094235 10.3389/fbioe.2018.00086 PMC6070628

[117] Tavakoli S , Klar AS . Advanced hydrogels as wound dressings. Biomolecules. 2020;10(8):1169. 32796593 10.3390/biom10081169 PMC7464761

[118] Kwee BJ , Lam J , Akue A , et al. Functional heterogeneity of IFN‐γicensed mesenchymal stromal cell immunosuppressive capacity on biomaterials. Proc Natl Acad Sci U S A. 2021;118(35):e2105972118. 34446555 10.1073/pnas.2105972118 PMC8536328

[119] Xiao S , Xiao C , Miao Y , et al. Human acellular amniotic membrane incorporating exosomes from adipose‐derived mesenchymal stem cells promotes diabetic wound healing. Stem Cell Res Ther. 2021;12(1):1‐16. 33926555 10.1186/s13287-021-02333-6 PMC8082232

[120] Golchin A , Shams F , Basiri A , et al. Combination therapy of stem cell‐derived exosomes and biomaterials in the wound healing. Stem Cell Rev Reports. 2022;18(6):1892‐1911. 10.1007/s12015-021-10309-5 35080745

[121] Ma S , Hu H , Wu J , et al. Functional extracellular matrix hydrogel modified with MSC‐derived small extracellular vesicles for chronic wound healing. Cell Prolif. 2022;55(4):e13196. 35156747 10.1111/cpr.13196 PMC9055911

[122] Klimczak A , Hinc P , Krawczenko A , et al. HATMSC secreted factors in the hydrogel as a potential treatment for chronic wounds—In vitro study. Int J Mol Sci. 2021;22(22):12241. 34830121 10.3390/ijms222212241 PMC8618182

[123] Su N , Hao Y , Wang F , Hou W , Chen H , Luo Y . Mesenchymal stromal exosome–functionalized scaffolds induce innate and adaptive immunomodulatory responses toward tissue repair. Sci Adv. 2021;7(20):7207‐7219. 10.1126/sciadv.abf7207 PMC8115917 33980490

[124] Kakabadze Z , Chakhunashvili D , Gogilashvili K , et al. Bone marrow stem cell and decellularized human amniotic membrane for the treatment of nonhealing wound after radiation therapy. Exp Clin Transplant. 2019;17(Suppl 1):92‐98. 30777530 10.6002/ect.MESOT2018.O29

